# Exploring the molecular characteristics of inflammatory bowel disease from the perspective of hypoxia-related genes

**DOI:** 10.3389/fphar.2025.1612676

**Published:** 2025-07-23

**Authors:** Lei Zheng, Lin Mou, Lingying Hao, Rui Chen, Yifei Wang, Miao Yu, Xueyan Zhang

**Affiliations:** ^1^Department of Gastroenterology, Harbin First Hospital, Harbin, Heilongjiang, China; ^2^Department of Neurology, Harbin First Hospital, Harbin, Heilongjiang, China; ^3^Department of Gastroenterology, The Second Affiliated Hospital of Harbin Medical University, Harbin, Heilongjiang, China; ^4^Department of Medical, Harbin First Hospital, Harbin, Heilongjiang, China

**Keywords:** inflammatory bowel disease, hypoxia, immune cells, macrophages, diagnosis model

## Abstract

**Background:**

Inflammatory bowel disease (IBD) constitutes a chronic inflammatory disorder affecting the gastrointestinal tract, characterized by a multifaceted pathogenesis that encompasses genetic, environmental, and immunological influences. The role of hypoxia in IBD pathophysiology has been recognized. However, the specific genes associated with hypoxia and their potential for diagnostic application remain inadequately investigated.

**Methods:**

Three datasets (GSE48958, GSE75214, and GSE179285) were procured from the Gene Expression Omnibus (GEO) database through the GEOquery package, all sourced from human colon tissue. Hypoxia-related genes (HRGs) were extracted from the GeneCards database. Data preprocessing involved mitigating batch effects *via* the sva package and normalizing with the limma package. The differential expression analysis, conducted with limma, uncovered 475 differentially expressed genes (DEGs), comprising 152 downregulated and 323 upregulated genes. A subset of 23 hypoxia-related differentially expressed genes (HRDEGs), including ADM, BHLHE40, CCL2, and CD274, was identified by intersecting DEGs with HRG sets.

**Results:**

The analysis identified 475 DEGs within the aggregated dataset, with 323 exhibiting upregulation and 152 downregulation. Enrichment analysis highlighted the significant role of these HRDEGs in critical processes such as angiogenesis and the HIF-1 signaling pathway. A diagnostic model (DM) integrating 13 HRDEGs exhibited high accuracy, achieving an area under the curve (AUC) exceeding 0.9 across various datasets. Immune infiltration analysis revealed substantial disparities in 13 distinct immune cell populations when comparing high-risk and low-risk cohorts.

**Conclusions:**

In summary, this investigation underscores the pivotal function of HRGs in IBD's pathogenesis and introduces a reliable DM grounded in these genetic factors. The findings accentuate the relevance of hypoxia-responsive pathways in IBD and enhance understanding of immune cell dynamics across differing risk profiles. Subsequent investigations should seek to confirm these biomarkers in clinical contexts and investigate therapeutic strategies targeting hypoxia-related pathways for effective IBD management.

## 1 Introduction

Inflammatory bowel disease (IBD), encompassing Crohn’s disease (CD) and ulcerative colitis (UC), represents a chronic and relapsing inflammatory disorder impacting the gastrointestinal tract. The worldwide incidence of IBD is escalating, with roughly 6.8 million individuals diagnosed globally in 2017 (Collaborators, 2020). This condition primarily affects young adults and is often associated with complex pathogenesis, including immunosuppression and hypoxia (Cooney et al., 2024). Characterized by recurrent inflammatory episodes, IBD manifests with manifestations encompassing abdominal pain, diarrhea, weight loss, and fatigue. Despite advancements in therapeutic options, including biologics and immunomodulators, many patients fail to achieve sustained remission, emphasizing the necessity for enhanced diagnostic and therapeutic strategies (Ghosh et al., 2020). Current diagnostic methods, predominantly reliant on endoscopic and histological evaluations, are invasive and may not accurately represent the molecular mechanisms underlying the disease (Lamb et al., 2019). Thus, the urgent need for novel biomarkers and less invasive diagnostic tools for effective IBD management is apparent.

Hypoxia, defined by low oxygen availability, has been associated with the onset of diverse inflammatory disorders, encompassing IBD. Hypoxia-inducible factors (HIFs) serve as key regulators of cellular responses under low oxygen conditions and play a significant role in modulating inflammatory pathways (Taylor and Colgan, 2017). Previous research has indicated that hypoxia exacerbates intestinal inflammation by promoting pro-inflammatory cytokine expression and enhancing immune cell infiltration (ICI) (Bhat and Rieder, 2022). Elevated levels of HIF-1α have been detected in the inflamed mucosa of individuals suffering from IBD, indicating a potential involvement of hypoxia signaling in disease progression (Karhausen et al., 2004). However, the varying expression of hypoxia-related genes (HRGs) in IBD and their diagnostic implications remain insufficiently explored. Inflammation and hypoxia frequently coexist in various pathological immune contexts, including chronic inflammatory and ischemic tissues (Taylor and Colgan, 2017). Additionally, obesity induces hypoxia in adipose tissue and the small intestine, stabilizing and activating HIF-1α and HIF-2α pathways, which can lead to adverse metabolic outcomes, encompassing insulin resistance and non-alcoholic fatty liver disease (Gonzalez et al., 2018). Furthermore, hypoxic-activated prodrugs have been extensively studied for targeting hypoxic tumor cells (Singleton et al., 2021).

This research investigates the varied expression of HRGs in IBD and assesses their potential diagnostic significance. Three datasets (GSE48958, GSE75214, and GSE179285) were procured from the GEO database and integrated employing the R package GEOquery. Following batch effect (BE) correction, a comprehensive dataset of 408 IBD samples and 61 control samples was obtained. Differentially expressed genes (DEGs) were ascertained utilizing the limma package, succeeded by functional enrichment analyses to elucidate the biological processes (BPs) and pathways linked to these genes. A diagnostic model (DM) was developed employing logistic regression, SVM, and LASSO regression, and its performance was validated with ROC curves and AUC values. ICI in high-risk and low-risk cohorts was analyzed employing the CIBERSORT algorithm, and a regulatory network involving transcription factors (TFs) and miRNAs was established.

The findings reveal significant differential expression of HRGs in IBD, highlighting their potential as diagnostic biomarkers. The DM developed demonstrated robust performance, suggesting that integrating HRGs into clinical practice could enhance IBD diagnostic accuracy. Moreover, the observed differences in ICI between high-risk and low-risk cohorts offer insights into the immunopathogenesis of IBD and may inform the development of targeted therapies. Additionally, the regulatory network analysis identifies key TFs and miRNAs that may influence HRG expression in IBD, paving the way for novel therapeutic interventions.

## 2 Methods and materials

### 2.1 Data downloading

Employing the R package GEOquery (Davis, 2007) (Version 2.66.0), datasets linked to IBD were procured from the GEO database (Barrett et al., 2013) (https://www.ncbi.nlm.nih.gov/geo/), specifically GSE48958 (Van der Goten et al., 2014), GSE75214 (Vancamelbeke et al., 2017), and GSE179285 (Keir et al., 2021). All specimens in these datasets were procured from *Homo sapiens*, with the colon as the tissue source. The chip platforms for GSE48958 and GSE75214 were both GPL6244, while GSE179285 utilized the GPL6480 platform. Comprehensive information regarding these datasets is depicted in Supplementary Table S3. GSE48958 comprised 13 IBD specimens and 8 control specimens, GSE75214 included 172 IBD specimens and 22 controls, and GSE179285 contained 223 IBD specimens and 31 control specimens. All IBD and control specimens from these datasets were incorporated into the analysis.

To compile a collection of HRGs, the GeneCards database (Stelzer et al., 2016) (https://www.genecards.org/) was utilized. This database offers extensive information on human genes. Using “Hypoxia” as the query keyword and applying filters for HRGs classified as “Protein Coding” with a relevance score greater than 5, a total of 152 HRGs were identified. The precise designations of these hypoxia-linked genes are enumerated in Supplementary Table S4.

The R package sva (Leek et al., 2012) (Version 3.50.0) facilitated the removal of BEs from datasets GSE48958, GSE75214, and GSE179285, yielding a unified GEO dataset. Afterward, the R package limma (Ritchie et al., 2015) (Version 3.54.2) was employed for standardization, probe annotation, and normalization of the combined dataset. Principal Component Analysis (PCA) (Ben Salem and Ben Abdelaziz, 2021) was executed to visualize the expression matrix prior to and following BE correction, transforming the data into low-dimensional representations for presentation in 2D or 3D graphs. Ultimately, the combined dataset encompassed 408 IBD samples and 61 controls.

### 2.2 DEGs linked to IBD and hypoxia

Utilizing the sample classification in the merged dataset, specimens were classified into IBD and control cohorts. Differential gene examination between IBD (Ritchie et al., 2015) and control samples was executed employing the R package limma (Ritchie et al., 2015) (Version 3.54.2). The parameters for identifying DEGs were established as |logFC| > 0.5 and adjusted p-value (adj.p) < 0.05. Genes exhibiting logFC >0.5 and adj.p < 0.05 were labeled as upregulated DEGs, whereas those with logFC < −0.5 and adj.p < 0.05 were designated as downregulated genes. Results from the differential analysis were illustrated utilizing a volcano plot generated with the R package ggplot2 (Version 3.4.4).

To pinpoint hypoxia-related differentially expressed genes (HRDEGs) linked to IBD, the DEGs fulfilling the parameters of |logFC| > 0.5 and adj.p < 0.05 were cross-referenced with HRGs, and a Venn diagram was created to illustrate the intersection.

### 2.3 Gene ontology (GO) and kyoto encyclopedia of genes and genomes (KEGG) enrichment analyses

GO analysis (Mi et al., 2019), including BP, Cellular Component (CC), and Molecular Function (MF), alongside KEGG pathway analysis (Kanehisa and Goto, 2000), was executed on the identified HRDEGs employing the R package clusterProfiler (Yu et al., 2012) (Version 4.4.4). The selection parameters for enrichment evaluation were established with adj.p < 0.05 and a false discovery rate (FDR) value (q-value) < 0.25, with p-value corrections applied utilizing the Benjamini-Hochberg (BH) method.

### 2.4 Gene set enrichment analysis (GSEA)

Additionally, GSEA (Subramanian et al., 2005) was executed on the genes from the merged GEO datasets. Genes were initially ordered based on their logFC values. The R package clusterProfiler (Yu et al., 2012) (Version 4.4.4) facilitated the GSEA on all genes within the combined GEO datasets. The parameters for GSEA included a seed of 2022, 1,000 permutations, a lower threshold of 10 genes per gene set, and an upper limit of 500 genes per gene set. The Molecular Signatures Database (MSigDB) (Liberzon et al., 2011) was utilized for this analysis. GSEA outcomes were filtered based on adj.p < 0.05 and FDR value (q-value) < 0.25, with p-values corrected employing the BH method.

### 2.5 Establishment of the DM for IBD

To develop DMs for IBD utilizing the merged GEO datasets, logistic regression analysis (LRA) was conducted on HRDEGs. This analysis explored the link between independent variables and the binary outcome variable, distinguishing IBD samples from control samples. Genes exhibiting a *P*-value below 0.05 were considered significant, forming the basis of the logistic regression model. A Forest Plot was employed to illustrate the expression patterns of the HRDEGs included in the model. Subsequently, Support Vector Machine (SVM) (Sanz et al., 2018) analysis was applied to further filter HRDEGs from those identified in the logistic regression model. Concurrently, LASSO (Least Absolute Shrinkage and Selection Operator) regression analysis (Engebretsen and Bohlin, 2019) was executed utilizing the R package glmnet (Version 4.1–8), with parameters set at 1,000 iterations and family = “binomial.” LASSO was employed to reduce overfitting and enhance model generalization by incorporating a penalty component (lambda × absolute value of the slope). The outcomes were depicted using DM graphs and variable trajectory plots.

The HRDEGs identified through LASSO modeling and SVM examination were cross-referenced via Venn diagramming to determine the ultimate cohort of model-specific genes. The IBD DM and corresponding RiskScore were subsequently derived by combining the expression levels of these model genes in the consolidated GEO datasets with the coefficients from the LASSO regression model. The RiskScore was computed utilizing the following formula:

$$\text{RiskScore}=\sum_{i}Coefficient\;\left({gene}_{i}\right)*mRNA\;Expression\;\left({gene}_{i}\right)$$

### 2.6 Evaluation of DMs for IBD

A Nomogram (Wu et al., 2020) was developed utilizing the R package rms (Version 6.7–1) to visually represent the link between model genes grounded in the LRA. Decision Curve Analysis (DCA) (Van Calster et al., 2018) was executed utilizing the R package ggDCA (Version 1.2) to evaluate the clinical value of the RiskScore in the combined GEO datasets. DCA serves as a direct method for appraising predictive models in clinical settings, diagnostic procedures, and molecular indicators.

The diagnostic performance of the RiskScore for IBD was further evaluated by plotting ROC curves and calculating the Area Under the Curve (AUC) utilizing the R package pROC (Version 1.18.5) (Robin et al., 2011). IBD specimens were classified into high-risk and low-risk cohorts grounded in the median RiskScore value from the IBD DM. Additionally, ROC curves were generated to evaluate the diagnostic value of the model genes themselves, with AUC values ranging from 0.5 to 1. An AUC close to 1 signifies excellent diagnostic capability, with AUC values between 0.7 and 0.9 suggesting moderate accuracy, and values exceeding 0.9 indicating high diagnostic precision.

### 2.7 Examination of immune infiltration in high-risk and low-risk cohorts

CIBERSORT (Newman et al., 2015), leveraging linear support vector regression, was employed to deconvolute the transcriptome expression matrix, enabling the assessment of immune cell composition and quantity in mixed cell populations. Utilizing the LM22 feature gene matrix, data with immune cell enrichment scores exceeding zero were filtered, ultimately yielding the ICI matrix specific to IBD specimens from the integrated GEO datasets. The R package ggplot2 was then utilized to create group comparison plots, depicting the differential expression of LM22 immune cells between high-risk and low-risk IBD cohorts. Subsequently, immune cells demonstrating notable disparities between these two cohorts were identified for additional examination. Correlations among immune cells were computed utilizing the Spearman algorithm, with results visualized through a correlation heatmap created via the R package pheatmap. Additionally, correlations between model genes and immune cells were determined based on the Spearman method, retaining only those with a P-value <0.05. The R package ggplot2 was then utilized to create a correlation bubble plot to represent the relationships between model genes and immune cells.

### 2.8 Development of regulatory network

Regulatory proteins known as TFs modulate gene expression through interactions with model genes at the post-transcriptional stage. The ChIPBase database (Zhou et al., 2017) (http://rna.sysu.edu.cn/chipbase/) was utilized to identify TFs associated with model genes and to analyze their regulatory roles. The mRNA-TF regulatory network was visualized utilizing Cytoscape software.

miRNAs, vital regulators in organismal development and evolution, modulate diverse target genes, and a single target gene can be controlled by several miRNAs. To investigate the link between model genes and miRNAs, miRNAs linked to model genes were procured from the ENCORI database (Li et al., 2014). The mRNA-miRNA regulatory network (Shannon et al., 2003) was also depicted employing Cytoscape software.

### 2.9 Statistical analysis

All data processing and analyses were executed utilizing R software (Version 4.2.2). Unless stated otherwise, statistical significance for normally distributed variables was evaluated utilizing independent Student’s T-tests when comparing two cohorts. For variables not normally distributed, the Mann-Whitney U Test (Wilcoxon Rank Sum Test) was utilized. The Kruskal-Wallis test was applied for comparisons involving three or more cohorts. Spearman correlation analysis was applied to ascertain the correlation coefficient between diverse molecules. All statistical p-values were two-tailed unless specified otherwise, with p-values below 0.05 deemed statistically significant.

## 3 Results

### 3.1 Merging of IBD datasets

**FIGURE 1:**
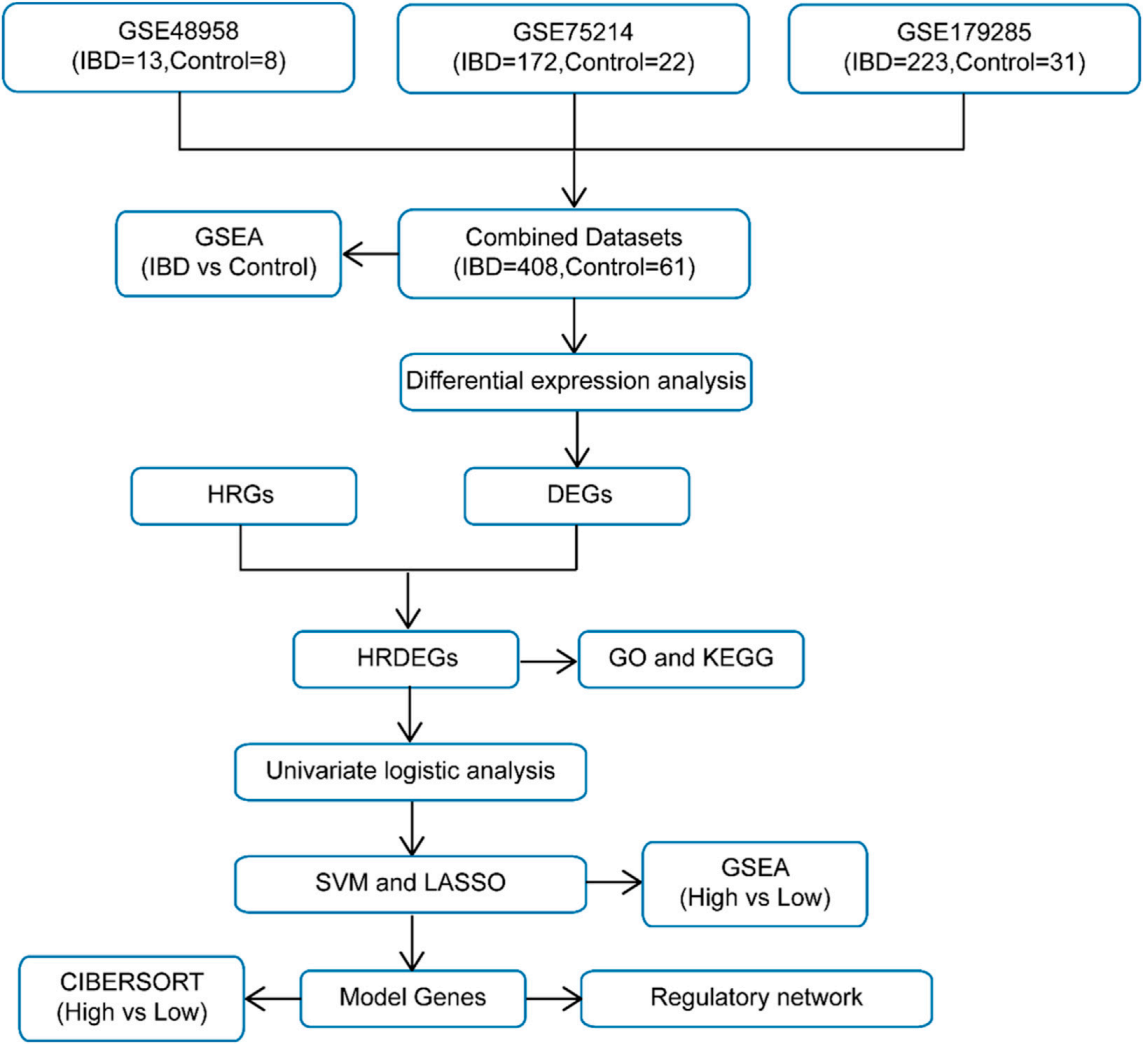
Technology roadmap.

The schematic roadmap of our study is illustrated in Figure 1. Firstly, the R package sva was employed to eliminate BEs from the IBD datasets GSE48958, GSE75214, and GSE179285, generating the combined datasets. A distribution boxplot (Supplementary Figures S1A,B) was then employed to contrast the expression values prior to and following BE removal. Additionally, a PCA plot (Supplementary Figures S1C, D) was applied to evaluate the dispersion of low-dimensional characteristics pre- and post-BE correction. Both the distribution boxplot and PCA plot demonstrated that BEs were effectively mitigated in the IBD datasets following the removal process.

### 3.2 DEGs related to IBD-associated hypoxia

**FIGURE 2:**
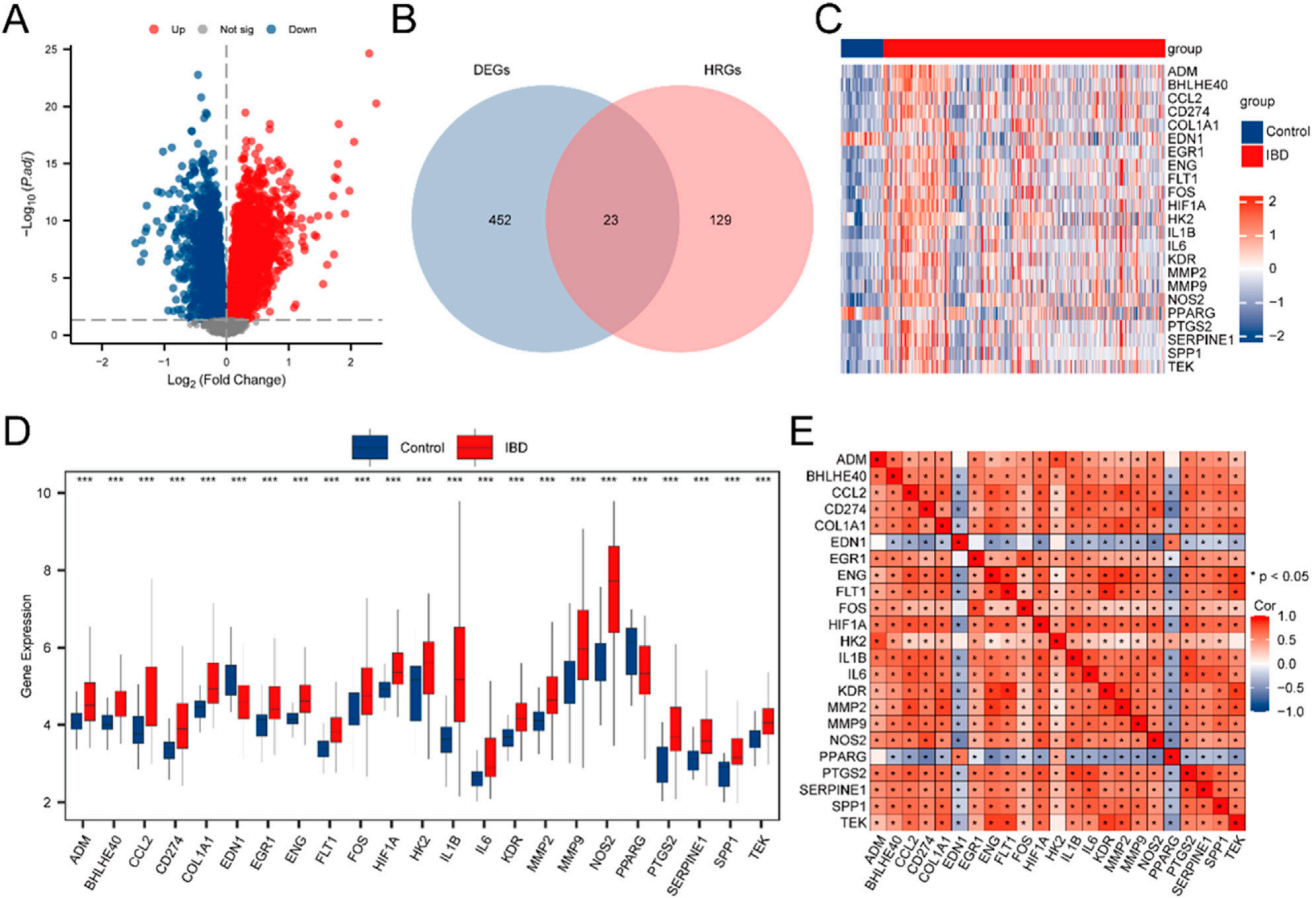
Differential Gene Expression Analysis. **(A)** Volcano plot illustrating the differential expression analysis of IBD samples versus Control samples in the Combined GEO Datasets. **(B)** Venn diagram displaying the intersection between DEGs and HRGs in the integrated GEO Datasets (Combined Datasets). **(C)** Heatmap depicting the expression of HRDEGs across sample cohorts in the unified GEO Datasets. **(D)** Group comparison plot of HRDEGs between different sample cohorts within the unified GEO Datasets (Combined Datasets). **(E)** Correlation heatmap of HRDEGs, showing gene-gene relationships. IBD samples are shown in red, and Control (Control) samples are shown in blue. In the heat map, red denotes high expression and blue denotes low expression. In the group comparison map, the symbol ** is equivalent to P < 0.01, signifying high statistical significance. *** denotes a p-value <0.001, signifying high statistical significance.

The Merged GEO Datasets were classified into IBD and Control samples. To assess gene expression differences between these cohorts, differential expression analysis was executed employing the R package limma, identifying DEGs. The examination uncovered 475 DEGs satisfying the criteria of |logFC| > 0.5 and adj.p < 0.05. Among these, 323 genes exhibited upregulated (logFC >0.5, adj.p < 0.05), while 152 exhibited downregulated (logFC < −0.5, adj.p < 0.05). The outcomes are depicted in a volcano plot (Figure 2A).

To identify HRDEGs, a Venn diagram was constructed to depict the overlap between DEGs and HRGs (Figure 2B), yielding 23 HRDEGs: ADM, BHLHE40, CCL2, CD274, COL1A1, EDN1, EGR1, ENG, FLT1, FOS, HIF1A, HK2, IL1B, IL6, KDR, MMP2, MMP9, NOS2, PPARG, PTGS2, SERPINE1, SPP1, and TEK. These HRDEGs were further analyzed and visualized through a heatmap produced utilizing the R package pheatmap, reflecting their expression differences in the dataset (Figure 2C).

For a deeper exploration of HRDEG expression in the consolidated GEO Datasets, a group comparison figure (Figure 2D) was produced to illustrate the differential expression of these 23 HRDEGs between IBD and Control samples. Significant expression differences were observed, with most HRDEGs (ADM, BHLHE40, CCL2, CD274, COL1A1, EGR1, ENG, FLT1, FOS, HIF1A, HK2, IL1B, IL6, KDR, MMP2, MMP9, NOS2, PTGS2, SERPINE1, SPP1, and TEK) being upregulated in IBD samples, while EDN1 and PPARG were downregulated. Additionally, a correlation heatmap was produced for the 23 HRDEGs (Figure 2E), indicating that most exhibited significant positive correlations.

### 3.3 GO and KEGG pathway enrichment analyses of hypoxia-related DEGs in IBD

GO and KEGG pathway enrichment analyses were executed to investigate the associations between BP, CC, MF, and KEGG pathways for the 23 HRDEGs in relation to IBD. Detailed results are depicted in Supplementary Table S5. The analysis suggested that these 23 HRDEGs were primarily concentrated in BP, such as modulation of angiogenesis, vasculature development, response to oxygen levels, response to lipopolysaccharide, and response to hypoxia. For CC, significant enrichment was observed in the endoplasmic reticulum lumen, among others. Regarding MF, the genes were linked to signaling receptor activator activity, cytokine activity, transmembrane receptor protein kinase activity, growth factor binding, and cytokine receptor binding. Moreover, the HRDEGs were notably enriched in several KEGG pathways, encompassing the HIF-1 signaling cascade, TNF signaling cascade, IL-17 signaling cascade, MAPK signaling cascade, and the IBD pathway. The outcomes of the GO and KEGG pathway enrichment analyses were depicted utilizing bubble plots (Supplementary Figure S2A). Additionally, loop network diagrams were created to illustrate the links between BP, CC, MF, and KEGG pathways (Supplementary Figures S2B–E), where the lines indicate links between molecules and the respective annotated terms. Larger nodes represent entries containing a higher number of molecules.

### 3.4 GSEA among different cohorts in combined GEO datasets

**FIGURE 3:**
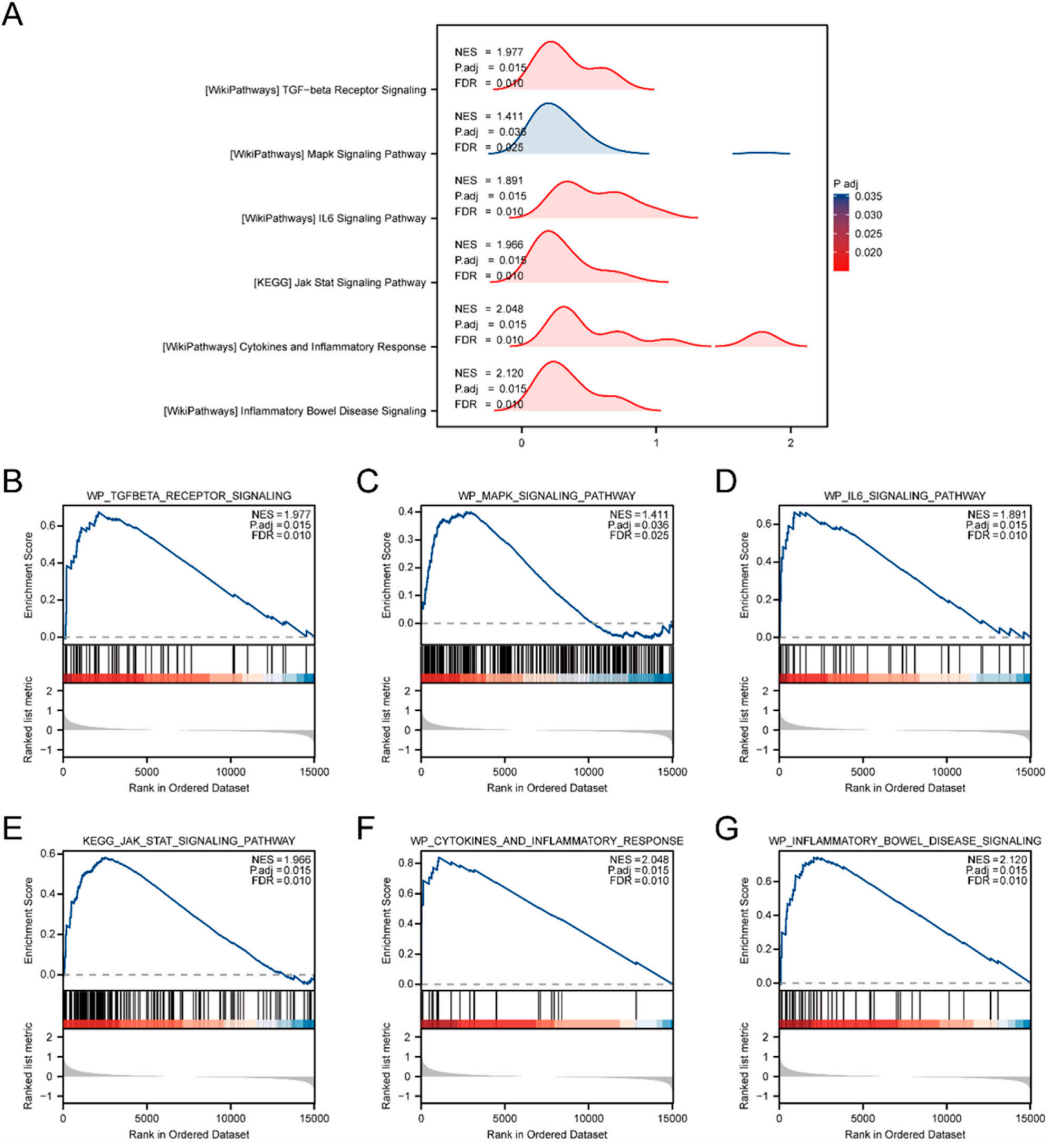
Combined Datasets analysis between IBD and Control cohorts using GSEA. **(A)** Mountain plot illustrating six key biological functions identified through GSEA in the IBD cohort relative to the Control cohort within the Combined GEO Datasets. **(B–G)** GSEA results showing marked enrichment of all genes in the following pathways: WP_TGFBETA_RECEPTOR_SIGNALING **(B)**, WP_MAPK_SIGNALING_PATHWAY **(C)**, WP_IL6_SIGNALING_PATHWAY **(D)**, KEGG_JAK_STAT_SIGNALING_PATHWAY **(E)**, WP_CYTOKINES_AND_INFLAMMATORY_RESPONSE **(F)**, and WP_INFLAMMATORY_BOWEL_DISEASE_SIGNALING **(G)**. GSEA. The analysis applied criteria of adj.p < 0.05 and FDR (q value) < 0.25 for significance.

To assess the influence of gene expression levels across all genes in the merged GEO Datasets (Combined Datasets) on IBD, GSEA was utilized. This approach examined the expression of all genes and the BPs, CCs, and MFs they influence (Figure 3A). Comprehensive findings are provided in Supplementary Table S6.

The examination uncovered substantial enhancement of genes in multiple pathways, encompassing the TGF-beta receptor signaling cascade (Figure 3B), MAPK signaling cascade (Figure 3C), and IL6 signaling cascade (Figure 3D). Additionally, the JAK-STAT signaling pathway (Figure 3E), cytokine and inflammatory response (Figure 3F), and the IBD signaling pathway (Figure 3G) were notably enriched, indicating their critical roles in biological functions and signaling processes related to IBD.

### 3.5 Establishment of the DM for IBD

**FIGURE 4:**
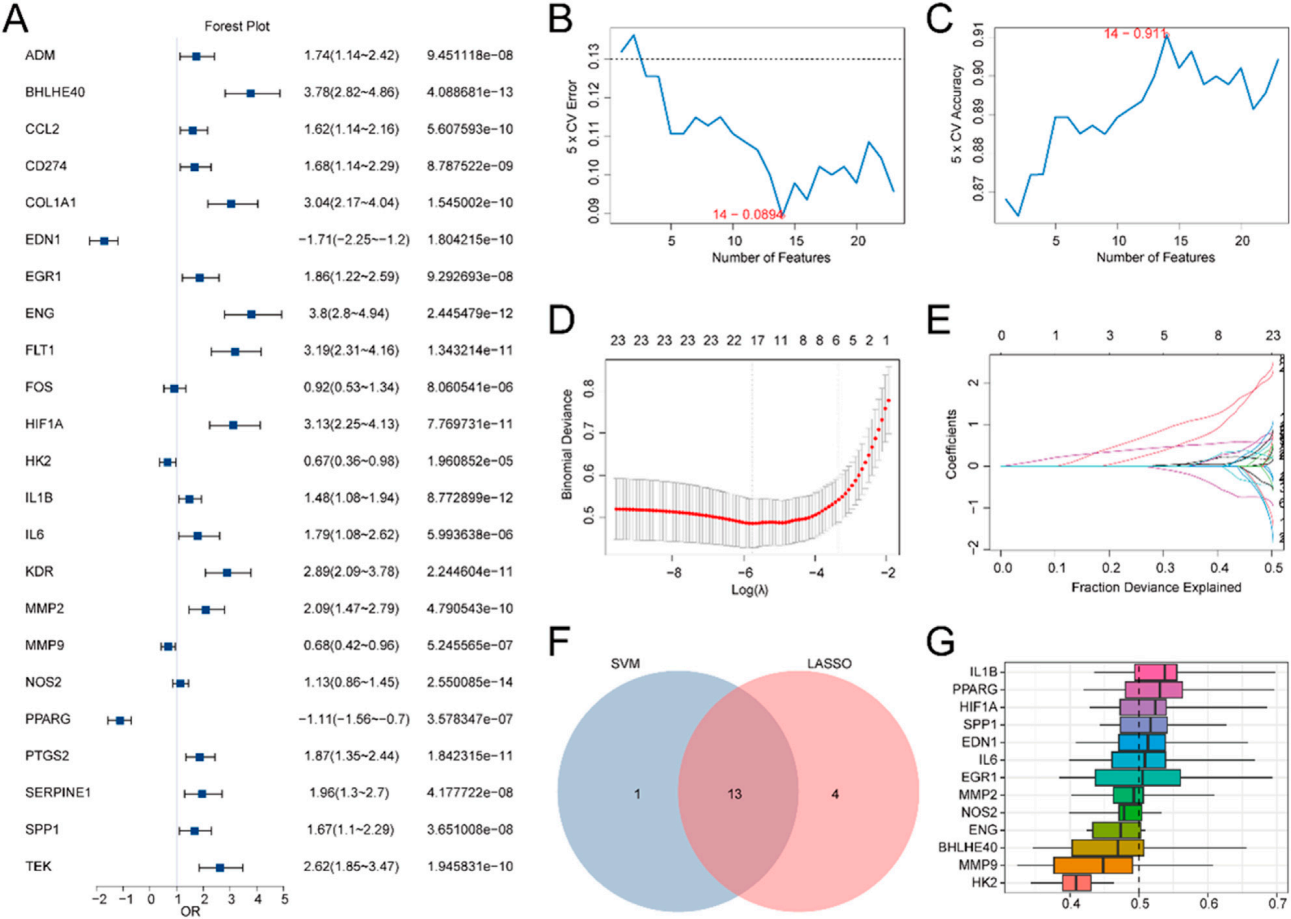
DM for IBD. **(A)** Forest plot illustrating the LRA of 23 HRDEGs. B-C. Visualization of the gene count yields the lowest error rate **(B)** and the highest accuracy **(C)** in the SVM algorithm. **(D,E)** DM plot **(D)** and variable trajectory plot **(E)** of the LASSO regression model. **(F)** Venn diagram showcasing the overlap between HRDEGs included in the LASSO regression model and those encompassed in the SVM model. **(G)** Boxplot showing the functional similarity analysis of model genes.

To evaluate the diagnostic potential of 23 HRDEGs in IBD, logistic regression was first applied to the dataset. A logistic regression model was built and depicted utilizing a Forest Plot (Figure 4A), demonstrating that all 23 HRDEGs were statistically significant (p < 0.05).

Subsequently, an SVM (Support Vector Machine) algorithm was utilized to develop an SVM model, building upon the logistic regression model. The model was optimized to minimize the error rate (Figure 4B) and maximize accuracy (Figure 4C). The analysis indicated that the SVM model reached its peak performance when using 14 HRDEGs, which were: BHLHE40, EDN1, EGR1, ENG, HIF1A, HK2, IL1B, IL6, MMP2, MMP9, NOS2, PPARG, PTGS2, and SPP1.

Additionally, a LASSO (Least Absolute Shrinkage and Selection Operator) regression model was created utilizing the same 23 HRDEGs from the logistic regression model. The LASSO regression plot (Figure 4D) and variable trajectory diagram (Figure 4E) provided a visual representation of the analysis, showing that 17 HRDEGs were retained in the LASSO model. These genes included: ADM, BHLHE40, COL1A1, EDN1, EGR1, ENG, FLT1, HIF1A, HK2, IL1B, IL6, MMP2, MMP9, NOS2, PPARG, SPP1, and TEK.

Next, the HRDEGs identified from both the LASSO regression model and the SVM model were juxtaposed utilizing a Venn diagram (Figure 4F), yielding 13 overlapping model genes: BHLHE40, EDN1, EGR1, ENG, HIF1A, HK2, IL1B, IL6, MMP2, MMP9, NOS2, PPARG, and SPP1. A DM for IBD was then constructed by integrating the expression levels of these 13 model genes with the regression coefficients from the LASSO model, resulting in the following RiskScore formula: RiskScore=BHLHE40*1.880263188+EDN1*−0.739172108+EGR1* 0.194906128+ENG*1.914504876+HIF1A*−0.473313989 +HK2* 0.59283215+IL1B* 0.354144093+IL6 *−0.421088826 +MMP2*0.455838964+MMP9*−0.406781925+NOS2* 0.578376745+PPARG * 0.250999791+SPP1 * 0.545610438 .

Ultimately, the R package GOSemSim was utilized to compute the GO terms for these 13 model genes. A boxplot (Figure 4G) was generated to illustrate the semantic resemblance among gene products and gene clusters. The results indicated that IL1B demonstrated the greatest functional affinity with other model genes.

### 3.6 Validation of DMs for IBD

**FIGURE 5:**
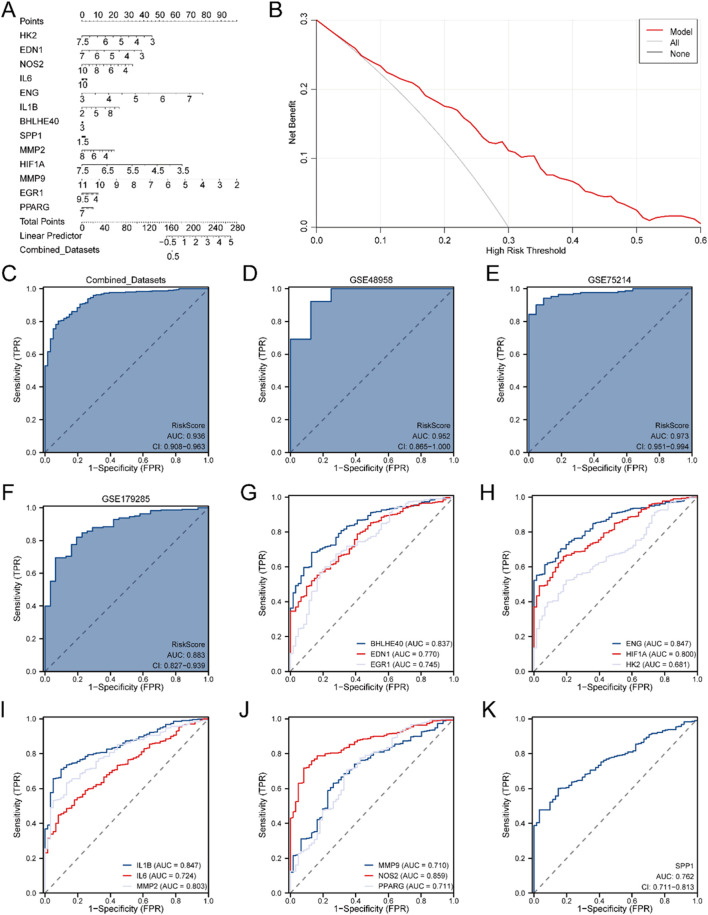
Diagnostic and Validation Analyses of IBD. **(A)** Nomogram depicting the impact of Model Genes on the DM for IBD in the Combined GEO Datasets. **(B)** DCA plot for assessing the clinical applicability of the IBD DM. **(C)** ROC curve for the RiskScore in the Combined GEO Datasets, evaluating diagnostic accuracy. **(D)** Diagnostic ROC curve of the RiskScore for the GSE48958 dataset. **(E)** Diagnostic ROC curve for the RiskScore in the GSE75214 dataset. **(F)** Diagnostic ROC curve for the RiskScore in the GSE179285 dataset. **(G)** Diagnostic ROC curves for BHLHE40, EDN1, and EGR1 in the Merged GEO Datasets. **(H)** Diagnostic ROC curves for ENG, HIF1A, and HK2 in the Merged GEO Datasets. **(I)** Diagnostic ROC curves for IL1B, IL6, and MMP2 in the Merged GEO Datasets. **(J)** Diagnostic ROC curves for MMP9, NOS2, and PPARG in the Merged GEO Datasets. **(K)** Diagnostic ROC curve for SPP1 in the Merged GEO Datasets. An AUC >0.5 indicates a molecule’s diagnostic potential, with AUC values closer to 1 representing superior diagnostic efficacy. AUC values between 0.5 and 0.7 suggest low accuracy, 0.7 to 0.9 indicate moderate accuracy, and AUC values above 0.9 indicate high accuracy.

To further substantiate the IBD DM’s efficacy, a Nomogram was generated utilizing the model genes to illustrate their relationships within the Combined GEO Datasets (Figure 5A). The results highlighted that MMP9 had significantly greater diagnostic utility than the other variables in the model.

Next, DCA was conducted to evaluate the clinical applicability of the IBD DM derived from the Combined GEO Datasets (Figure 5B). The DCA results demonstrated that the model’s performance line remained consistently higher than the “All Positive” and “All Negative” lines within a certain range, suggesting a superior net benefit and overall effectiveness. Additionally, the R package pROC was employed to generate an ROC curve grounded in the RiskScore from the Merged GEO Datasets. The ROC curve (Figure 5C) revealed that the RiskScore exhibited high diagnostic accuracy (AUC >0.9) across different cohorts.

Further validation was performed by calculating the RiskScore for the GSE48958 (Figure 5D), GSE75214 (Figure 5E), and GSE179285 (Figure 5F) datasets, followed by ROC curve analysis. The results suggested that the RiskScore for the GSE48958 and GSE75214 datasets had high accuracy across diverse cohorts (AUC >0.9), while the GSE179285 dataset indicated moderate accuracy (0.7 < AUC <0.9).

Finally, ROC curves were generated utilizing the R package pROC to evaluate the expression levels of the model genes in the Combined GEO Datasets. The ROC curves (Figures 5G–K) indicated that 12 model genes—BHLHE40, EDN1, EGR1, ENG, HIF1A, IL1B, IL6, MMP2, MMP9, NOS2, PPARG, and SPP1—indicated moderate accuracy (0.7 < AUC <0.9) between different cohorts, while HK2 exhibited lower accuracy (0.5 < AUC <0.7).

### 3.7 GSEA between high- and low-risk cohorts in merged GEO datasets

Firstly, IBD specimens were classified into high-risk and low-risk cohorts grounded in the median LASSO risk score (RiskScore) from the IBD DM. To explore the influence of gene expression levels on the high and low risk of developing IBD, GSEA was conducted. This analysis examined the link between the expression levels of all genes in IBD specimens and their associated BPs, CCs, and MFs. The outcomes are illustrated in a mountain plot (Supplementary Figure S3A), and specific details are provided in Supplementary Table S7. The examination uncovered that gene expression in IBD samples was significantly enriched in several key pathways, including reactome cellular response to hypoxia (Supplementary Figure S3B), TGF-beta receptor signaling (Supplementary Figure S3C), JAK-STAT signaling pathway (Supplementary Figure S3D), TH17 cell differentiation pathway (Supplementary Figure S3E), IBD signaling (Supplementary Figure S3F), and the intestinal immune network for IgA production (Supplementary Figure S3G), along with other biologically pertinent functions and signaling cascades.

### 3.8 Immune infiltration analysis between high- and low-risk cohorts

**FIGURE 6:**
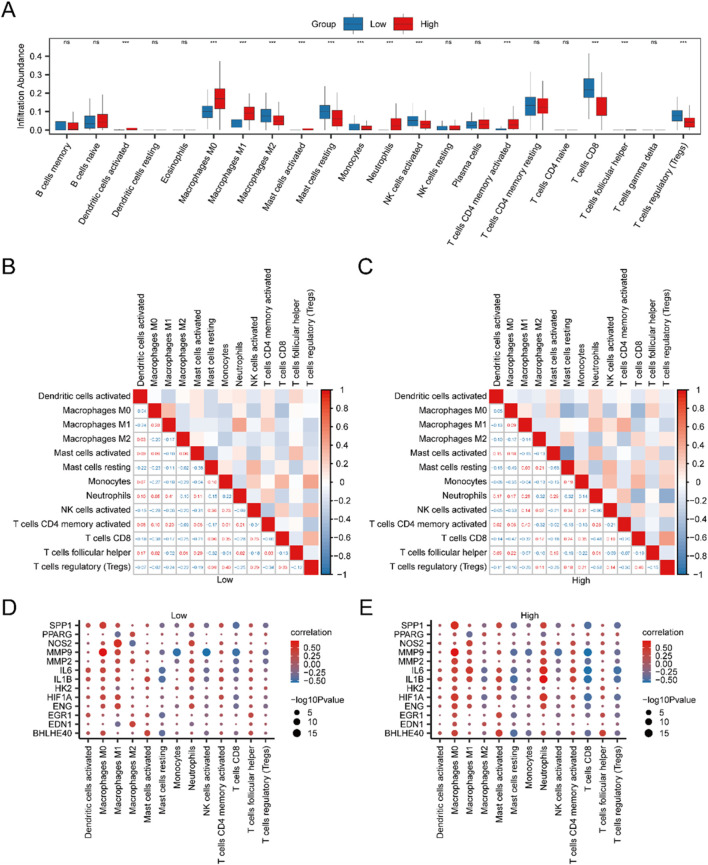
Risk Cohort Immune Infiltration Analysis using the CIBERSORT Algorithm. **(A)** Group comparison of ICI between high-risk (High) and low-risk (Low) cohorts of IBD specimen. **(B,C)** Correlation analysis outcomes of ICI abundance in low-risk (Low) **(B)** and high-risk (High) **(C)** IBD samples. **(D,E)** Bubble plots depicting the correlation between ICI abundance and model genes in low-risk **(D)** and high-risk **(E)** IBD samples. The examination utilized single-sample Gene-Set Enrichment Analysis (ssGSEA). In the plots, “ns” indicates a p-value ≥0.05 (not statistically significant), while “***” indicates p-values <0.001, indicating high statistical significance. Red indicates the high-risk (High) cohort, and blue indicates the low-risk (Low) cohort of IBD samples.

The CIBERSORT algorithm was utilized to evaluate the infiltration levels of 22 distinct immune cell populations within high- and low-risk IBD cohorts, employing IBD specimens from the Merged GEO Datasets. A group comparison plot visualized the disparities in ICI between the two cohorts. As shown in Figure 6A, 13 immune cells displayed statistically significant differences (p < 0.05), including activated dendritic cells, M0 macrophages, M1 macrophages, M2 macrophages, activated mast cells, resting mast cells, monocytes, neutrophils, activated NK cells, activated CD4^+^ T cells, CD8^+^ T cells, T follicular helper cells (Tfh), and regulatory T cells (Tregs). A correlation heatmap further illustrated the relationships between the infiltration levels of these 13 immune cells across the high- and low-risk cohorts in the immune infiltration analysis (Figures 6B,C). Finally, the link between model genes and ICI was depicted through correlation bubble plots (Figures 6D,E). MMP9 demonstrated the most robust positive association with M0 macrophages in both the low-risk cohort (r = 0.705, p < 0.05) and the high-risk cohort (r = 0.720, p < 0.05).

### 3.9 Construction of protein-protein interaction network

**FIGURE 7:**
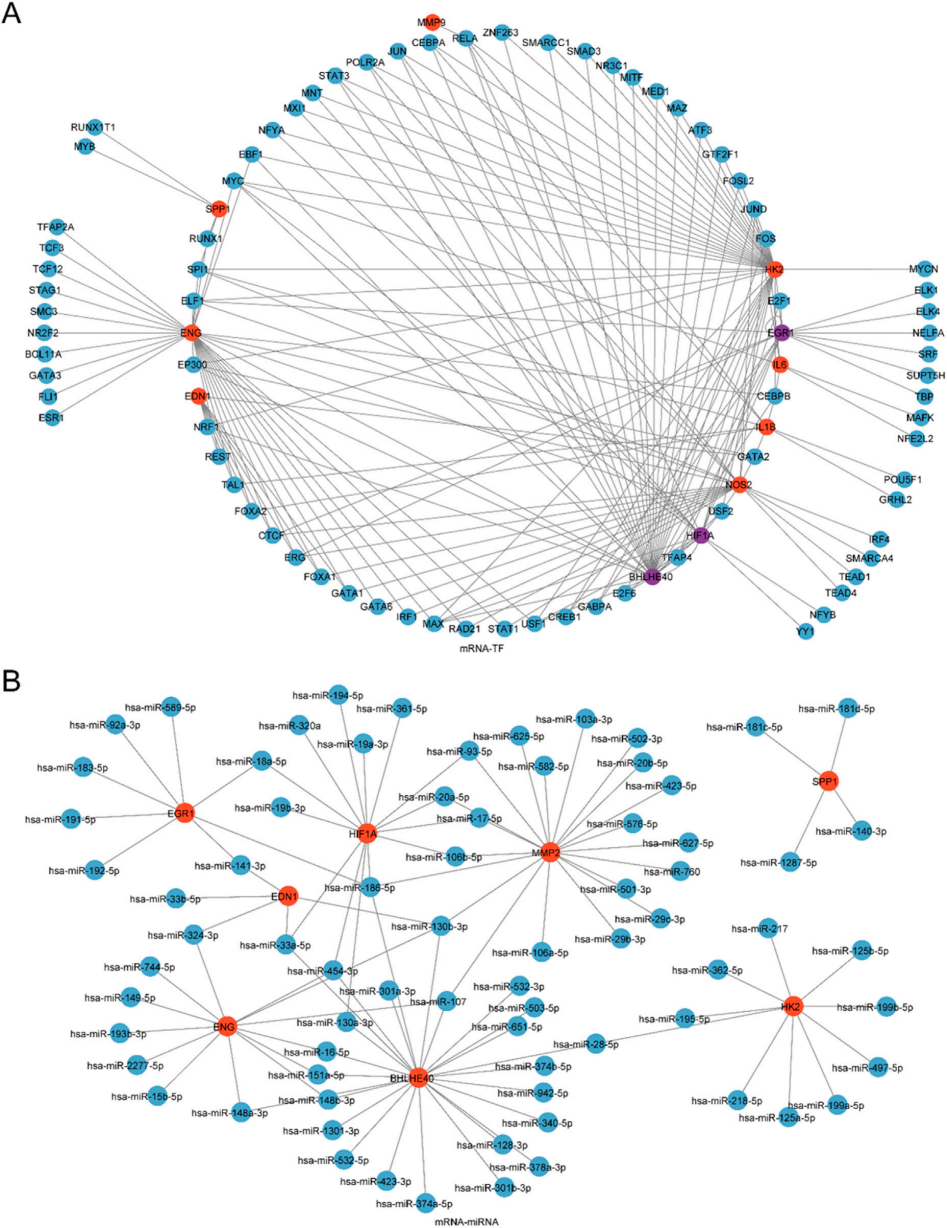
Regulatory Network of Model Genes. **(A)** mRNA-miRNA Regulatory Network of model genes, where red nodes indicate mRNAs and blue nodes indicate miRNAs. **(B)** mRNA-TF Regulatory Network of model genes, where red nodes indicate mRNAs, blue nodes denote TFs, and purple nodes indicate genes that function as both mRNA and TF.

TFs interacting with the model genes were ascertained employing the ChIPBase database, and an mRNA-TF regulatory network was generated and rendered with Cytoscape software (Figure 7A). This network encompassed 11 model genes and 80 TFs, with BHLHE40, EGR1, and HIF1A serving as both model genes and TFs. Comprehensive information is depicted in Supplementary Table S1.

Next, miRNAs linked to the model genes were ascertained through the ENCORI database, and an mRNA-miRNA regulatory network was created and depicted utilizing Cytoscape software (Figure 7B). This network comprised 8 model genes and 74 miRNAs, with specific details listed in Supplementary Table S2.

## 4 Discussion

IBD represents a chronic, relapsing inflammatory disorder affecting the gastrointestinal tract, chiefly comprising CD and UC. It severely impacts patients' quality of life, manifesting with indicators like abdominal pain, diarrhea, and weight loss, while posing risks for serious complications like colorectal cancer and intestinal strictures (Xavier and Podolsky, 2007). The disease places a considerable strain on individuals, healthcare systems, and society, with notable morbidity and an elevated risk of colorectal cancer (Kappelman, 2013). Despite therapeutic advances, including biologics and small molecule therapies, numerous patients struggle to attain enduring symptom relief, highlighting the pressing demand for improved diagnostic approaches and treatment modalities.

This study centers on the varied expression of HRGs in IBD and their potential diagnostic relevance, alongside ICI examination in high- and low-risk patient cohorts. Hypoxia plays a pivotal role in IBD pathophysiology, driving various cellular processes that exacerbate the inflammatory response (Colgan and Taylor, 2010). Through the integration of multiple datasets and advanced bioinformatics techniques, this research aims to identify key HRGs and develop a robust DM. The findings offer deeper insights into the molecular mechanisms of IBD, potentially leading to innovative diagnostic and therapeutic approaches that can improve patient outcomes.

Adrenomedullin (ADM), a multifunctional peptide, is implicated in vasodilation, angiogenesis, and anti-inflammatory responses. In the context of IBD, ADM appears to have a protective role, modulating inflammation and preserving intestinal barrier integrity. Elevated ADM levels in patients with IBD suggest its involvement in the disease’s pathogenesis, potentially mediated through the HIF-1 signaling pathways, which are active under the hypoxic conditions typical of inflamed tissues (Ashizuka et al., 2021). The marked upregulation of ADM noted in this investigation highlights its potential as a diagnostic indicator for IBD and as a therapeutic focus for reducing inflammation and promoting mucosal healing.

BHLHE40 (alternatively referred to as DEC1) is a TF regulating circadian rhythms and cellular hypoxic responses. Its role in IBD is linked to immune modulation and inflammatory control. BHLHE40 is induced under hypoxic conditions, which are prevalent in inflamed intestinal tissues and have been implicated in the regulation of pro-inflammatory cytokines (Zafar et al., 2021). Moreover, studies have found that BHLHE40 inhibits cell proliferation and angiogenesis under hypoxic conditions. The deficiency of BHLHE40 extensively limits the inflammatory signaling pathways, hypoxic responses, and glycolytic gene expression in macrophages (Acosta-Iborra et al., 2024). The upregulation of BHLHE40 in IBD samples highlighted in this study emphasizes its potential as a diagnostic marker and its role in hypoxia-driven inflammatory pathways. Targeting BHLHE40 may offer promising therapeutic opportunities for managing inflammation in patients with IBD.

CCL2, also known as monocyte chemoattractant protein-1 (MCP-1), functions as a chemokine that attracts monocytes, memory T cells, and dendritic cells to areas of inflammation. In IBD, elevated levels of CCL2 promote the ICIs into the intestinal mucosa, intensifying inflammation and tissue damage (Mello et al., 2021). Studies have shown that CCL2 promotes the selective activation of peritoneal macrophages, thereby enhancing the production of reactive oxygen species in the intestinal tract of mice (Yu et al., 2022). The marked upregulation of CCL2 observed in this study aligns with its established role in amplifying inflammatory responses in IBD, suggesting it is a prospective focus for treatment strategies aimed at reducing ICI and curbing inflammation.

CD274 (PD-L1), an immune checkpoint protein, serves a vital function in modulating immune responses and maintaining tolerance, with implications for chronic inflammatory diseases. In IBD, PD-L1 expression on intestinal epithelial and immune cells may modulate the local immune milieu, potentially sustaining chronic inflammation (Chulkina et al., 2020; Nguyen et al., 2022). Our findings of altered CD274 expression in IBD samples suggest its involvement in the immunopathogenesis of the disease. Targeting the PD-1/PD-L1 pathway could offer therapeutic benefits by restoring immune balance and reducing inflammatory responses in patients with IBD. These findings align with prior research demonstrating CD274s association with the dysregulation of refractory IBD immune responses and increased expression in certain immune cells.

HIF1A, a critical TF, mediates cellular adaptation to hypoxia by regulating genes related to angiogenesis, metabolism, and inflammation. In the context of IBD, HIF1A activation is essential for adapting to the hypoxic microenvironment in inflamed tissues (Colgan and Taylor, 2010). HIF (hypoxia-inducible factor) can affect inflammatory bowel disease (IBD) through multiple mechanisms under hypoxic conditions: When hypoxia occurs, the stability of HIF-1α/2α is enhanced and downstream genes are activated. On the one hand, it promotes the expression of intestinal epithelial tight junction proteins to strengthen the mucosal barrier and reduce bacterial translocation. On the other hand, it inhibits the activation of pro-inflammatory cells such as Th1 and Th17 and promotes the functions of anti-inflammatory cells such as Treg and M2-type macrophages (Jiang et al., 2014). At the same time, induce the expression of glycolysois-related enzymes to provide energy for cells and maintain intestinal homeostasis (Gucev et al., 2018); However, the expression of HIF in the intestinal mucosa of IBD patients is often decreased. Its abnormal function or absence can weaken the protective and immunomodulatory abilities of the intestinal barrier (Muenchau et al., 2019), thereby participating in the occurrence and development of IBD. The activation of HIF in animal models has also been confirmed to alleviate intestinal inflammation (Campbell et al., 2014). The downregulation of HIF1A observed in this study may reflect a disrupted hypoxic response in patients with IBD. Modulating HIF1A activity could present therapeutic opportunities by improving the adaptive response to hypoxia and mitigating inflammation.

Matrix metallopeptidase 2 (MMP2) is an enzyme implicated in the degradation of extracellular matrix components and tissue remodeling. In IBD, MMP2 expression is linked to processes of tissue damage and repair (Vandenbroucke and Libert, 2014). The upregulation of MMP2 in our study indicates its role in inflammatory and tissue remodeling responses in IBD. Targeting MMP2 activity could offer therapeutic advantages by regulating tissue repair processes and reducing inflammation in affected patients.

The enrichment analysis of the 23 HRDEGs highlighted their involvement in key BPs and pathways, such as the regulation of angiogenesis, response to oxygen levels, the HIF-1 signaling cascade, and the TNF signaling cascade. These pathways play pivotal roles in IBD pathogenesis, emphasizing the mechanisms contributing to the disease’s pathology.

The HIF-1 signaling pathway is a well-established mediator of cellular adaptation to hypoxia, a hallmark of the inflamed intestinal mucosa in patients with IBD. HIFs regulate genes that are critical for processes like angiogenesis, metabolism, and immune response, thereby playing a pivotal role in maintaining the intestinal barrier and modulating inflammation (Villareal and Xue, 2024). As a TF, HIF-1α facilitates cellular adaptation to low oxygen levels by translocating into the nucleus, where it forms a dimer with HIF-1β and attaches to hypoxia-responsive sequences in target genes. This mechanism is vital for supporting gut barrier function and modulating the inflammatory response. Some studies have utilized the hypoxic mouse colitis model induced by dextran sulfate sodium (DSS), confirming that hypoxia can regulate the HIF-1α signaling pathway by up-regulating lipocalin 2 (LCN2), affecting the glycolysis process and driving the polarization of M1 macrophages, thereby aggravating the pathological damage of colitis (Yang et al., 2024).

Similarly, the TNF signaling pathway is a key driver of inflammation in IBD. Tumor necrosis factor (TNF), a pro-inflammatory cytokine, triggers inflammatory cascades that result in tissue damage and chronic gut inflammation (Atreya and Neurath, 2008). In recent years, monoclonal antibodies targeting TNF-alpha, such as infliximab, have been widely used to treat Crohn’s disease, demonstrating effectiveness in reducing inflammation (Belaiche and Louis, 2000). The enrichment of HRDEGs in the TNF signaling pathway suggests that these genes could be contributing to the heightened inflammatory state in IBD, supporting their possible diagnostic biomarkers or therapeutic targets. However, the intersection of TNF signaling with hypoxia in IBD remains underexplored, warranting further investigation.

In summary, the enrichment of HRDEGs in critical pathways such as HIF-1 and TNF signaling underscores their significant functions in the pathophysiology of IBD. These pathways offer an essential understanding of the molecular processes driving the disease and highlight promising targets for diagnosis and treatment.

The immune infiltration analysis, conducted using the CIBERSORT algorithm, revealed notable disparities in the abundance of 13 immune cell populations between high-risk and low-risk IBD cohorts. Among these, activated dendritic cells, M0 macrophages, and M1 macrophages displayed significant variation. Dendritic cells, as primary antigen-presenting cells, are essential in initiating and regulating adaptive immune responses. Their increased activation in IBD suggests enhanced immune surveillance and antigen presentation, potentially contributing to the persistent inflammation observed in patients with IBD (Steinman and Banchereau, 2007).

Macrophages, particularly the M0 and M1 subtypes, play critical roles in inflammation and tissue remodeling. M0 macrophages are undifferentiated and can polarize into either pro-inflammatory M1 or anti-inflammatory M2 macrophages, contingent upon environmental signals. The increased presence of M1 macrophages in high-risk patients with IBD suggests a shift towards a pro-inflammatory state known to exacerbate intestinal inflammation through the generation of cytokines like TNF-α, IL-1β, and IL-6 (Murray and Wynn, 2011). This pro-inflammatory environment can sustain the inflammatory cycle, leading to tissue damage and fibrosis, key features of IBD pathology. While our study identified significant differences between M0 and M1 macrophages, research on this topic remains limited, providing a potential avenue for subsequent explorations.

The observed patterns of differential ICI emphasize the intricate link between HRG expression and immune responses in IBD. Hypoxia, commonly present in inflamed tissues, influences immune cell function and differentiation. For instance, HIFs can modulate the behavior of macrophages and dendritic cells, thus affecting the overall inflammatory response. The significant enrichment of hypoxia-related pathways in high-risk cohorts, as revealed by GSEA, further underscores the interconnectedness of hypoxia and immune dynamics in IBD pathogenesis.

These findings highlight the therapeutic potential of targeting ICI and hypoxia pathways in IBD. Elucidating the particular functions and processes of immune cells in hypoxic environments could lead to the development of precise interventions aimed at modulating immune responses and reducing inflammation in patients with IBD. Subsequent investigations should concentrate on clarifying the molecular interplay between hypoxia and immune cells, potentially identifying novel therapeutic targets.

However, this study has certain limitations. Due to financial constraints and the limitations of our hospital laboratory, no wet lab experiments were conducted to biologically validate these findings. Moreover, despite the integration of three datasets, the comparatively limited sample size might restrict the applicability of our outcomes. The lack of clinical corroboration represents another notable constraint, as it is crucial for substantiating the diagnostic value of the detected biomarkers in medical environments. Furthermore, while the use of multiple datasets increased the sample size, it may have introduced BEs that could confound the results despite our efforts to correct these effects.

## 5 Conclusion

In conclusion, this study identified HRDEGs in IBD and demonstrated their possible diagnostic value. We constructed a robust DM and identified notable disparities in ICI between high- and low-risk cohorts. Additionally, we explored potential regulatory networks involving TFs and miRNAs. While these findings offer a strong basis for future research and clinical applications, further validation and investigation are necessary to fully realize their potential for improving IBD diagnosis and treatment.

## Data Availability

Publicly available datasets were analyzed in this study. This data can be found here: GEO database at https://www.ncbi.nlm.nih.gov/geo/, reference number GSE48958, GSE75214, and GSE179285.

## References

[B1] Acosta-Iborra B. Gil-Acero A. I. Sanz-Gómez M. Berrouayel Y. Puente-Santamaría L. Alieva M. (2024). Bhlhe40 regulates proliferation and angiogenesis in mouse embryoid bodies under hypoxia. Int. J. Mol. Sci. 25 (14), 7669. 10.3390/ijms25147669 39062912 PMC11277088

[B2] Ashizuka S. Kita T. Inatsu H. Kitamura K. (2021). Adrenomedullin: a novel therapeutic for the treatment of inflammatory bowel disease. Biomedicines 9 (8), 1068. 10.3390/biomedicines9081068 34440272 PMC8391925

[B3] Atreya R. Neurath M. F. (2008). Signaling molecules: the pathogenic role of the IL-6/STAT-3 trans signaling pathway in intestinal inflammation and in colonic cancer. Curr. Drug Targets 9 (5), 369–374. 10.2174/138945008784221116 18473764

[B4] Barrett T. Wilhite S. E. Ledoux P. Evangelista C. Kim I. F. Tomashevsky M. (2013). NCBI GEO: archive for functional genomics data sets--update. Nucleic Acids Res. 41 (Database issue), D991–D995. 10.1093/nar/gks1193 23193258 PMC3531084

[B5] Belaiche J. Louis E. (2000). Treatment of crohn disease in adults with tumor necrosis factor-alpha (TNF-alpha) antibodies. Rev. Med. Liege 55 (9), 827–832. 11105596

[B6] Ben Salem K. Ben Abdelaziz A. (2021). Principal component analysis (PCA). Tunis. Med. 99 (4), 383–389. 35244921 PMC8734479

[B7] Bhat S. Rieder F. (2022). Hypoxia-inducible factor 1-Alpha stabilizers in the treatment of inflammatory bowel diseases: oxygen as a novel IBD therapy? J. Crohns Colitis 16 (12), 1924–1932. 10.1093/ecco-jcc/jjac092 35776532 PMC10060721

[B8] Campbell E. L. Bruyninckx W. J. Kelly C. J. Glover L. E. McNamee E. N. Bowers B. E. (2014). Transmigrating neutrophils shape the mucosal microenvironment through localized oxygen depletion to influence resolution of inflammation. Immunity 40 (1), 66–77. 10.1016/j.immuni.2013.11.020 24412613 PMC3951457

[B9] Chulkina M. Beswick E. J. Pinchuk I. V. (2020). Role of PD-L1 in gut mucosa tolerance and chronic inflammation. Int. J. Mol. Sci. 21 (23), 9165. 10.3390/ijms21239165 33271941 PMC7730745

[B10] Colgan S. P. Taylor C. T. (2010). Hypoxia: an alarm signal during intestinal inflammation. Nat. Rev. Gastroenterol. Hepatol. 7 (5), 281–287. 10.1038/nrgastro.2010.39 20368740 PMC4077542

[B11] Collaborators G. B. D. I. B. D. (2020). The global, regional, and national burden of inflammatory bowel disease in 195 countries and territories, 1990-2017: a systematic analysis for the global burden of disease study 2017. Lancet Gastroenterol. Hepatol. 5 (1), 17–30. 10.1016/S2468-1253(19)30333-4 31648971 PMC7026709

[B12] Cooney R. Tang D. Barrett K. Russell R. K. (2024). Children and young adults with inflammatory bowel disease have an increased incidence and risk of developing mental health conditions: a UK population-based cohort study. Inflamm. Bowel Dis. 30 (8), 1264–1273. 10.1093/ibd/izad169 37603846 PMC11291622

[B13] Davis S. M. P. Meltzer P. S. (2007). GEOquery: a bridge between the gene expression omnibus (GEO) and BioConductor. Bioinformatics 23 (14), 1846–1847. 10.1093/bioinformatics/btm254 17496320

[B14] Engebretsen S. Bohlin J. (2019). Statistical predictions with glmnet. Clin. Epigenetics 11 (1), 123. 10.1186/s13148-019-0730-1 31443682 PMC6708235

[B15] Ghosh S. Sensky T. Casellas F. Rioux L. C. Ahmad T. Marquez J. R. (2020). A global, prospective, observational study measuring disease burden and suffering in patients with ulcerative colitis using the pictorial representation of illness and self-measure tool. J. Crohns Colitis 15 (2), 228–237. 10.1093/ecco-jcc/jjaa159 32722760 PMC7904086

[B16] Gonzalez F. J. Xie C. Jiang C. (2018). The role of hypoxia-inducible factors in metabolic diseases. Nat. Rev. Endocrinol. 15 (1), 21–32. 10.1038/s41574-018-0096-z 30275460 PMC6624429

[B17] Gucev Z. Kalcev G. Laban N. Saveski A. Z B. N P. (2018). Characteristic diagnostic clues of metatropic dysplasia: the lumbothoracic humpback with dumbbell appearance of the long bones. Balk. J. Med. Genet. 21 (2), 35–38. 10.2478/bjmg-2018-0025 PMC6454243 30984522

[B18] Jiang W. Su J. Zhang X. Cheng X. Zhou J. Shi R. (2014). Elevated levels of Th17 cells and Th17-related cytokines are associated with disease activity in patients with inflammatory bowel disease. Inflamm. Res. 63 (11), 943–950. 10.1007/s00011-014-0768-7 25129403

[B19] Kanehisa M. Goto S. (2000). KEGG: kyoto encyclopedia of genes and genomes. Nucleic Acids Res. 28 (1), 27–30. 10.1093/nar/28.1.27 10592173 PMC102409

[B20] Kappelman M. D. (2013). Environmental factors and inflammatory bowel disease: elusive or nonexistent? Inflamm. Bowel Dis. 19 (3), 548–549. 10.1097/MIB.0b013e318281ce99 23429447 PMC4361946

[B21] Karhausen J. Furuta G. T. Tomaszewski J. E. Johnson R. S. Colgan S. P. Haase V. H. (2004). Epithelial hypoxia-inducible factor-1 is protective in murine experimental colitis. J. Clin. Invest 114 (8), 1098–1106. 10.1172/JCI21086 15489957 PMC522241

[B22] Keir M. E. Fuh F. Ichikawa R. Acres M. Hackney J. A. Hulme G. (2021). Regulation and role of αE integrin and gut homing integrins in migration and retention of intestinal lymphocytes during inflammatory bowel disease. J. Immunol. 207 (9), 2245–2254. 10.4049/jimmunol.2100220 34561227 PMC8525869

[B23] Lamb C. A. Kennedy N. A. Raine T. Hendy P. A. Smith P. J. Limdi J. K. (2019). British society of gastroenterology consensus guidelines on the management of inflammatory bowel disease in adults. Gut 68 (Suppl. 3), s1–s106. 10.1136/gutjnl-2019-318484 31562236 PMC6872448

[B24] Leek J. T. Johnson W. E. Parker H. S. Jaffe A. E. Storey J. D. (2012). The sva package for removing batch effects and other unwanted variation in high-throughput experiments. Bioinformatics 28 (6), 882–883. 10.1093/bioinformatics/bts034 22257669 PMC3307112

[B25] Li J. H. Liu S. Zhou H. Qu L. H. Yang J. H. (2014). starBase v2.0: decoding miRNA-ceRNA, miRNA-ncRNA and protein-RNA interaction networks from large-scale CLIP-seq data. Nucleic Acids Res. 42 (Database issue), D92–D97. 10.1093/nar/gkt1248 24297251 PMC3964941

[B26] Liberzon A. Subramanian A. Pinchback R. Thorvaldsdottir H. Tamayo P. Mesirov J. P. (2011). Molecular signatures database (MSigDB) 3.0. Bioinformatics 27 (12), 1739–1740. 10.1093/bioinformatics/btr260 21546393 PMC3106198

[B27] Mello J. D. C. Gomes L. E. M. Silva J. F. Siqueira N. S. N. Pascoal L. B. Martinez C. A. R. (2021). The role of chemokines and adipokines as biomarkers of crohn's disease activity: a systematic review of the literature. Am. J. Transl. Res. 13 (8), 8561–8574. 34539979 PMC8430066

[B28] Mi H. Muruganujan A. Ebert D. Huang X. Thomas P. D. (2019). PANTHER version 14: more genomes, a new PANTHER GO-slim and improvements in enrichment analysis tools. Nucleic Acids Res. 47 (D1), D419-D426–d426. 10.1093/nar/gky1038 30407594 PMC6323939

[B29] Muenchau S. Deutsch R. de Castro I. J. Hielscher T. Heber N. Niesler B. (2019). Hypoxic environment promotes barrier formation in human intestinal epithelial cells through regulation of MicroRNA 320a expression. Mol. Cell Biol. 39 (14), e00553-18. 10.1128/MCB.00553-18 31061092 PMC6597885

[B30] Murray P. J. Wynn T. A. (2011). Protective and pathogenic functions of macrophage subsets. Nat. Rev. Immunol. 11 (11), 723–737. 10.1038/nri3073 21997792 PMC3422549

[B31] Newman A. M. Liu C. L. Green M. R. Gentles A. J. Feng W. Xu Y. (2015). Robust enumeration of cell subsets from tissue expression profiles. Nat. Methods 12 (5), 453–457. 10.1038/nmeth.3337 25822800 PMC4739640

[B32] Nguyen J. Finkelman B. S. Escobar D. Xue Y. Wolniak K. Pezhouh M. (2022). Overexpression of programmed death ligand 1 in refractory inflammatory bowel disease. Hum. Pathol. 126, 19–27. 10.1016/j.humpath.2022.04.011 35489437

[B33] Ritchie M. E. Phipson B. Wu D. Hu Y. Law C. W. Shi W. (2015). limma powers differential expression analyses for RNA-Sequencing and microarray studies. Nucleic Acids Res. 43 (7), e47. 10.1093/nar/gkv007 25605792 PMC4402510

[B34] Robin X. Turck N. Hainard A. Tiberti N. Lisacek F. Sanchez J. C. (2011). pROC: an open-source package for R and S+ to analyze and compare ROC curves. BMC Bioinforma. 12, 77. 10.1186/1471-2105-12-77 PMC3068975 21414208

[B35] Sanz H. Valim C. Vegas E. Oller J. M. Reverter F. (2018). SVM-RFE: selection and visualization of the Most relevant features through non-linear kernels. BMC Bioinforma. 19 (1), 432. 10.1186/s12859-018-2451-4 PMC6245920 30453885

[B36] Shannon P. Markiel A. Ozier O. Baliga N. S. Wang J. T. Ramage D. (2003). Cytoscape: a software environment for integrated models of biomolecular interaction networks. Genome Res. 13 (11), 2498–2504. 10.1101/gr.1239303 14597658 PMC403769

[B37] Singleton D. C. Macann A. Wilson W. R. (2021). Therapeutic targeting of the hypoxic tumour microenvironment. Nat. Rev. Clin. Oncol. 18 (12), 751–772. 10.1038/s41571-021-00539-4 34326502

[B38] Steinman R. M. Banchereau J. (2007). Taking dendritic cells into medicine. Nature 449 (7161), 419–426. 10.1038/nature06175 17898760

[B39] Stelzer G. Rosen N. Plaschkes I. Zimmerman S. Twik M. Fishilevich S. (2016). The GeneCards suite: from gene data mining to disease genome sequence analyses. Curr. Protoc. Bioinforma. 54 (1.30), 1. 31-31.30.33. 10.1002/cpbi.5 27322403

[B40] Subramanian A. Tamayo P. Mootha V. K. Mukherjee S. Ebert B. L. Gillette M. A. (2005). Gene set enrichment analysis: a knowledge-based approach for interpreting genome-wide expression profiles. Proc. Natl. Acad. Sci. U S A 102 (43), 15545–15550. 10.1073/pnas.0506580102 16199517 PMC1239896

[B41] Taylor C. T. Colgan S. P. (2017). Regulation of immunity and inflammation by hypoxia in immunological niches. Nat. Rev. Immunol. 17 (12), 774–785. 10.1038/nri.2017.103 28972206 PMC5799081

[B42] Van Calster B. Wynants L. Verbeek J. F. M. Verbakel J. Y. Christodoulou E. Vickers A. J. (2018). Reporting and interpreting decision curve analysis: a guide for investigators. Eur. Urol. 74 (6), 796–804. 10.1016/j.eururo.2018.08.038 30241973 PMC6261531

[B43] Vancamelbeke M. Vanuytsel T. Farré R. Verstockt S. Ferrante M. Van Assche G. (2017). Genetic and transcriptomic bases of intestinal epithelial barrier dysfunction in inflammatory bowel disease. Inflamm. Bowel Dis. 23 (10), 1718–1729. 10.1097/MIB.0000000000001246 28885228 PMC6461205

[B44] Vandenbroucke R. E. Libert C. (2014). Is there new hope for therapeutic matrix metalloproteinase inhibition? Nat. Rev. Drug Discov. 13 (12), 904–927. 10.1038/nrd4390 25376097

[B45] Van der Goten J. Vanhove W. Lemaire K. Van Lommel L. Machiels K. Wollants W. J. (2014). Integrated miRNA and mRNA expression profiling in inflamed Colon of patients with ulcerative colitis. PLoS One 9 (12), e116117. 10.1371/journal.pone.0116117 25546151 PMC4278881

[B46] Villareal L. B. Xue X. (2024). The emerging role of hypoxia and environmental factors in inflammatory bowel disease. Toxicol. Sci. 198 (2), 169–184. 10.1093/toxsci/kfae004 38200624 PMC10964750

[B47] Wu J. Zhang H. Li L. Hu M. Chen L. Xu B. (2020). A nomogram for predicting overall survival in patients with low-grade endometrial stromal sarcoma: a population-based analysis. Cancer Commun. (Lond) 40 (7), 301–312. 10.1002/cac2.12067 32558385 PMC7365459

[B48] Xavier R. J. Podolsky D. K. (2007). Unravelling the pathogenesis of inflammatory bowel disease. Nature 448 (7152), 427–434. 10.1038/nature06005 17653185

[B49] Yang Y. H. Yan F. Shi P. S. Yang L. C. Cui D. J. (2024). HIF-1α pathway orchestration by LCN2: a key player in hypoxia-mediated colitis exacerbation. Inflammation 47 (4), 1491–1519. 10.1007/s10753-024-01990-y 38819583

[B50] Yu G. Wang L. G. Han Y. He Q. Y. (2012). clusterProfiler: an R package for comparing biological themes among gene clusters. OMICS 16 (5), 284–287. 10.1089/omi.2011.0118 22455463 PMC3339379

[B51] Yu W. Zhang Y. Kang C. Zheng Y. Liu X. Liang Z. (2022). The pharmacological evidence of the chang-yan-ning formula in the treatment of colitis. Front. Pharmacol. 13, 1029088. 10.3389/fphar.2022.1029088 36278202 PMC9579319

[B52] Zafar A. Ng H. P. Kim G. D. Chan E. R. Mahabeleshwar G. H. (2021). BHLHE40 promotes macrophage pro-inflammatory gene expression and functions. FASEB J. 35 (10), e21940. 10.1096/fj.202100944R 34551158 PMC8607355

[B53] Zhou K. R. Liu S. Sun W. J. Zheng L. L. Zhou H. Yang J. H. (2017). ChIPBase v2.0: decoding transcriptional regulatory networks of non-coding RNAs and protein-coding genes from ChIP-seq data. Nucleic Acids Res. 45 (D1), D43-D50–d50. 10.1093/nar/gkw965 27924033 PMC5210649

