# Supplementary material for: Exploring the molecular characteristics of inflammatory bowel disease from the perspective of hypoxia-related genes
Source: Front Pharmacol. 2025 Jul 23;16:1612676. doi: 10.3389/fphar.2025.1612676 (PMC12325073; doi:10.3389/fphar.2025.1612676)
Supplement: Supplementary file 1 [file Supplementaryfile1.docx]

**Table S1. mRNA-TF interaction network nodes.**

| mRNA | TF |  | mRNA | TF |  | mRNA | TF |
| --- | --- | --- | --- | --- | --- | --- | --- |
| HK2 | E2F6 |  | EDN1 | REST |  | HIF1A | NFYB |
| HK2 | EBF1 |  | EDN1 | TAL1 |  | HIF1A | RELA |
| HK2 | EGR1 |  | EDN1 | CTCF |  | HIF1A | USF1 |
| HK2 | EP300 |  | EDN1 | ERG |  | HIF1A | USF2 |
| HK2 | FOS |  | EDN1 | FOXA1 |  | HIF1A | YY1 |
| HK2 | FOSL2 |  | EDN1 | FOXA2 |  | HIF1A | BHLHE40 |
| HK2 | GTF2F1 |  | EDN1 | GATA1 |  | HIF1A | E2F1 |
| HK2 | ATF3 |  | EDN1 | GATA2 |  | HIF1A | E2F6 |
| HK2 | HIF1A |  | EGR1 | CREB1 |  | HIF1A | EGR1 |
| HK2 | JUN |  | EGR1 | CTCF |  | HK2 | ELF1 |
| HK2 | JUND |  | EGR1 | E2F1 |  | HK2 | GABPA |
| HK2 | MAX |  | EGR1 | EGR1 |  | HK2 | MNT |
| HK2 | MAZ |  | EGR1 | ELF1 |  | HK2 | MXI1 |
| HK2 | MED1 |  | EGR1 | ELK1 |  | HK2 | MYCN |
| HK2 | MITF |  | EGR1 | ELK4 |  | HK2 | NFYA |
| HK2 | MYC |  | EGR1 | ERG |  | HK2 | SPI1 |
| HK2 | BHLHE40 |  | EGR1 | GABPA |  | HK2 | CREB1 |
| HK2 | NR3C1 |  | EGR1 | NELFA |  | IL1B | POU5F1 |
| HK2 | NRF1 |  | EGR1 | SRF |  | IL1B | RELA |
| HK2 | POLR2A |  | EGR1 | SUPT5H |  | IL1B | SPI1 |
| HK2 | RELA |  | EGR1 | TBP |  | IL1B | TAL1 |
| HK2 | SMAD3 |  | EGR1 | TFAP4 |  | IL1B | CEBPA |
| HK2 | SMARCC1 |  | ENG | EBF1 |  | IL1B | CEBPB |
| HK2 | STAT3 |  | ENG | ELF1 |  | IL1B | FOXA2 |
| HK2 | TFAP4 |  | ENG | EP300 |  | IL1B | GATA2 |
| HK2 | USF1 |  | ENG | ERG |  | IL1B | GRHL2 |
| HK2 | USF2 |  | ENG | ESR1 |  | IL6 | FOS |
| HK2 | CEBPA |  | ENG | FLI1 |  | IL6 | JUND |
| HK2 | CEBPB |  | ENG | FOXA1 |  | IL6 | MAFK |
| HK2 | ZNF263 |  | ENG | FOXA2 |  | IL6 | MYC |
| BHLHE40 | E2F6 |  | ENG | GATA1 |  | IL6 | NFE2L2 |
| BHLHE40 | EBF1 |  | ENG | GATA2 |  | IL6 | CEBPB |
| BHLHE40 | EGR1 |  | ENG | GATA3 |  | MMP9 | RELA |
| BHLHE40 | EP300 |  | ENG | GATA6 |  | NOS2 | CTCF |
| BHLHE40 | FOS |  | ENG | BCL11A |  | NOS2 | ELF1 |
| BHLHE40 | FOSL2 |  | ENG | IRF1 |  | NOS2 | EP300 |
| BHLHE40 | GTF2F1 |  | ENG | MAX |  | NOS2 | ERG |
| BHLHE40 | ATF3 |  | ENG | BHLHE40 |  | NOS2 | FOS |
| BHLHE40 | HIF1A |  | ENG | MYC |  | NOS2 | FOXA1 |
| BHLHE40 | JUN |  | ENG | NR2F2 |  | NOS2 | GATA1 |
| BHLHE40 | JUND |  | ENG | RAD21 |  | NOS2 | GATA2 |
| BHLHE40 | MAX |  | ENG | REST |  | NOS2 | GATA6 |
| BHLHE40 | MAZ |  | ENG | RUNX1 |  | NOS2 | IRF1 |
| BHLHE40 | MED1 |  | ENG | SMC3 |  | NOS2 | IRF4 |
| BHLHE40 | MITF |  | ENG | SPI1 |  | NOS2 | JUN |
| BHLHE40 | MYC |  | ENG | STAG1 |  | NOS2 | JUND |
| BHLHE40 | BHLHE40 |  | ENG | STAT1 |  | NOS2 | MAX |
| BHLHE40 | NR3C1 |  | ENG | TAL1 |  | NOS2 | POLR2A |
| BHLHE40 | NRF1 |  | ENG | TCF12 |  | NOS2 | RAD21 |
| BHLHE40 | POLR2A |  | ENG | TCF3 |  | NOS2 | SMARCA4 |
| BHLHE40 | RELA |  | ENG | TFAP2A |  | NOS2 | SPI1 |
| BHLHE40 | SMAD3 |  | ENG | USF1 |  | NOS2 | STAT1 |
| BHLHE40 | SMARCC1 |  | ENG | USF2 |  | NOS2 | STAT3 |
| BHLHE40 | STAT3 |  | ENG | CEBPB |  | NOS2 | TEAD1 |
| BHLHE40 | TFAP4 |  | ENG | CTCF |  | NOS2 | TEAD4 |
| BHLHE40 | USF1 |  | HIF1A | MAX |  | NOS2 | USF2 |
| BHLHE40 | USF2 |  | HIF1A | MNT |  | SPP1 | MYB |
| BHLHE40 | CEBPA |  | HIF1A | MXI1 |  | SPP1 | RUNX1 |
| BHLHE40 | CEBPB |  | HIF1A | MYC |  | SPP1 | RUNX1T1 |
| BHLHE40 | ZNF263 |  | HIF1A | NFYA |  | SPP1 | SPI1 |
| EDN1 | NRF1 |  |  |  |  |  |  |

TF: Transcription factors.

**Table S2. mRNA-miRNA interaction network nodes.**

| miRNA | mRNA |  | miRNA | mRNA |
| --- | --- | --- | --- | --- |
| hsa-miR-16-5p | BHLHE40 |  | hsa-miR-2277-5p | ENG |
| hsa-miR-28-5p | BHLHE40 |  | hsa-miR-17-5p | HIF1A |
| hsa-miR-33a-5p | BHLHE40 |  | hsa-miR-18a-5p | HIF1A |
| hsa-miR-107 | BHLHE40 |  | hsa-miR-19a-3p | HIF1A |
| hsa-miR-148a-3p | BHLHE40 |  | hsa-miR-19b-3p | HIF1A |
| hsa-miR-128-3p | BHLHE40 |  | hsa-miR-20a-5p | HIF1A |
| hsa-miR-130a-3p | BHLHE40 |  | hsa-miR-33a-5p | HIF1A |
| hsa-miR-186-5p | BHLHE40 |  | hsa-miR-93-5p | HIF1A |
| hsa-miR-301a-3p | BHLHE40 |  | hsa-miR-130a-3p | HIF1A |
| hsa-miR-130b-3p | BHLHE40 |  | hsa-miR-186-5p | HIF1A |
| hsa-miR-374a-5p | BHLHE40 |  | hsa-miR-194-5p | HIF1A |
| hsa-miR-378a-3p | BHLHE40 |  | hsa-miR-320a | HIF1A |
| hsa-miR-148b-3p | BHLHE40 |  | hsa-miR-106b-5p | HIF1A |
| hsa-miR-423-3p | BHLHE40 |  | hsa-miR-361-5p | HIF1A |
| hsa-miR-503-5p | BHLHE40 |  | hsa-miR-454-3p | HIF1A |
| hsa-miR-532-5p | BHLHE40 |  | hsa-miR-28-5p | HK2 |
| hsa-miR-651-5p | BHLHE40 |  | hsa-miR-199a-5p | HK2 |
| hsa-miR-454-3p | BHLHE40 |  | hsa-miR-199b-5p | HK2 |
| hsa-miR-340-5p | BHLHE40 |  | hsa-miR-217 | HK2 |
| hsa-miR-151a-5p | BHLHE40 |  | hsa-miR-218-5p | HK2 |
| hsa-miR-532-3p | BHLHE40 |  | hsa-miR-125b-5p | HK2 |
| hsa-miR-374b-5p | BHLHE40 |  | hsa-miR-125a-5p | HK2 |
| hsa-miR-301b-3p | BHLHE40 |  | hsa-miR-195-5p | HK2 |
| hsa-miR-942-5p | BHLHE40 |  | hsa-miR-362-5p | HK2 |
| hsa-miR-1301-3p | BHLHE40 |  | hsa-miR-497-5p | HK2 |
| hsa-miR-33a-5p | EDN1 |  | hsa-miR-17-5p | MMP2 |
| hsa-miR-141-3p | EDN1 |  | hsa-miR-20a-5p | MMP2 |
| hsa-miR-130b-3p | EDN1 |  | hsa-miR-93-5p | MMP2 |
| hsa-miR-324-3p | EDN1 |  | hsa-miR-29b-3p | MMP2 |
| hsa-miR-33b-5p | EDN1 |  | hsa-miR-103a-3p | MMP2 |
| hsa-miR-18a-5p | EGR1 |  | hsa-miR-106a-5p | MMP2 |
| hsa-miR-92a-3p | EGR1 |  | hsa-miR-107 | MMP2 |
| hsa-miR-192-5p | EGR1 |  | hsa-miR-186-5p | MMP2 |
| hsa-miR-183-5p | EGR1 |  | hsa-miR-106b-5p | MMP2 |
| hsa-miR-141-3p | EGR1 |  | hsa-miR-29c-3p | MMP2 |
| hsa-miR-191-5p | EGR1 |  | hsa-miR-130b-3p | MMP2 |
| hsa-miR-186-5p | EGR1 |  | hsa-miR-20b-5p | MMP2 |
| hsa-miR-589-5p | EGR1 |  | hsa-miR-576-5p | MMP2 |
| hsa-miR-16-5p | ENG |  | hsa-miR-582-5p | MMP2 |
| hsa-miR-107 | ENG |  | hsa-miR-625-5p | MMP2 |
| hsa-miR-148a-3p | ENG |  | hsa-miR-627-5p | MMP2 |
| hsa-miR-15b-5p | ENG |  | hsa-miR-423-5p | MMP2 |
| hsa-miR-149-5p | ENG |  | hsa-miR-501-3p | MMP2 |
| hsa-miR-130b-3p | ENG |  | hsa-miR-502-3p | MMP2 |
| hsa-miR-148b-3p | ENG |  | hsa-miR-760 | MMP2 |
| hsa-miR-324-3p | ENG |  | hsa-miR-181c-5p | SPP1 |
| hsa-miR-193b-3p | ENG |  | hsa-miR-181d-5p | SPP1 |
| hsa-miR-454-3p | ENG |  | hsa-miR-140-3p | SPP1 |
| hsa-miR-151a-5p | ENG |  | hsa-miR-1287-5p | SPP1 |
| hsa-miR-744-5p | ENG |  |  |  |

**Table S3. IBD Datasets Information list.**

|  | GSE48958 | GSE75214 | GSE179285 |
| --- | --- | --- | --- |
| Platform | GPL6244 | GPL6244 | GPL6480 |
| Species | Homo sapiens | Homo sapiens | Homo sapiens |
| Tissue | colon | colon | colon |
| Samples in IBD group | 13 | 172 | 223 |
| Samples in Control group | 8 | 22 | 31 |
| Reference | Integrated miRNA and mRNA expression profiling in inflamed colon of patients with ulcerative colitis | Genetic and Transcriptomic Bases of Intestinal Epithelial Barrier Dysfunction in Inflammatory Bowel Disease | Regulation and Role of αE Integrin and Gut Homing Integrins in Migration and Retention of Intestinal Lymphocytes during Inflammatory Bowel Disease |

IBD,Inflammatory Bowel Disease.

**Table S4. List of HRGs.**

| HRGs | HRGs | HRGs | HRGs |
| --- | --- | --- | --- |
| HIF1A | KDR | CYB5R3 | ALKBH5 |
| EGLN1 | CITED2 | MMP2 | MDM2 |
| EGLN3 | STAT3 | SIRT1 | LDHA |
| HIF3A | NOS2 | PIK3CG | NOX4 |
| VEGFA | CXCL8 | RORA | TLR4 |
| EGLN2 | PDK1 | SDHB | BMPR2 |
| EPAS1 | COL1A1 | REST | SRC |
| HIF1AN | DDIT4 | SP1 | FGF2 |
| VHL | PTGS2 | SIAH2 | RAC1 |
| ARNT | ADM | NOS1 | SPP1 |
| SETD2 | BCL2 | ELOB | BHLHE40 |
| HYOU1 | IL1B | RWDD3 | RBX1 |
| HILPDA | CUL2 | ANGPTL4 | CRP |
| HIGD1A | NFKB1 | SFTPC | LRP5 |
| EPO | HSP90AA1 | NFE2L2 | IFNG |
| CA9 | SESN2 | VEGFC | MAPK8 |
| HIGD2A | EGFR | CXCL12 | LIMD1 |
| EP300 | PGF | FAM162A | PKM |
| HIGD1B | NOS3 | ANGPT2 | DPP4 |
| TP53 | ELOC | EGR1 | RHOA |
| P4HTM | CASP9 | EPOR | PPARG |
| SLC2A1 | JAK2 | MMP9 | SOX9 |
| HIGD1C | CCL2 | MGARP | PRKAA1 |
| HIGD2B | SERPINE1 | RELA | TERT |
| BNIP3 | CXCR4 | TH | ARNT2 |
| MTOR | MYC | PSMA7 | PRKCA |
| MAPK1 | NDRG1 | BAX | HMGB1 |
| HMOX1 | ACE | NOTCH1 | CDH1 |
| JUN | BDNF | ENG | THBS1 |
| IL6 | MAPK3 | PRKAA2 | LOXL2 |
| TNF | SOD1 | PIK3CA | SOD2 |
| CTNNB1 | HIPK2 | PFKFB4 | FUNDC1 |
| CASP3 | TIGAR | HGF | AHR |
| TGFB1 | CCN2 | XDH | MIF |
| CREBBP | FLT1 | TEK | IL10 |
| CREB1 | MAPK14 | HK2 | ENO1 |
| EDN1 | CD274 | FOS | IGF1 |
| AKT1 | LEP | PTEN | ATF4 |

HRGs,Hypoxia-Related Genes.

**Table S5. GO and KEGG enrichment analysis results.**

| ONTOLOGY | | ID | Description | BgRatio | p.adjust | qvalue | Count |
| --- | --- | --- | --- | --- | --- | --- | --- |
| BP | GO:0045765 | | regulation of angiogenesis | 345/18800 | 9.07 e-11 | 2.84 e-11 | 11 |
| BP | GO:1901342 | | regulation of vasculature development | 351/18800 | 9.07 e-11 | 2.84 e-11 | 11 |
| BP | GO:0070482 | | response to oxygen levels | 324/18800 | 7.53 e-10 | 2.36 e-10 | 10 |
| BP | GO:0032496 | | response to lipopolysaccharide | 333/18800 | 8.24 e-10 | 2.58 e-10 | 10 |
| BP | GO:0001666 | | response to hypoxia | 286/18800 | 6.51 e-09 | 2.04 e-09 | 9 |
| CC | GO:0005788 | | endoplasmic reticulum lumen | 311/19594 | 0.001947 | 0.001557 | 5 |
| KEGG | hsa04066 | | HIF-1 signaling pathway | 109/8164 | 1.53 e-08 | 8.42 e-09 | 8 |
| KEGG | hsa04668 | | TNF signaling pathway | 112/8164 | 5.19 e-07 | 2.87 e-07 | 7 |
| KEGG | hsa04657 | | IL-17 signaling pathway | 94/8164 | 3.04 e-06 | 1.68 e-06 | 6 |
| KEGG | hsa04010 | | MAPK signaling pathway | 294/8164 | 0.006335 | 0.003497 | 5 |
| KEGG | hsa05321 | | Inflammatory bowel disease | 65/8164 | 0.042293 | 0.023349 | 2 |
| MF | GO:0030546 | | signaling receptor activator activity | 496/18410 | 0.000569 | 0.000249 | 6 |
| MF | GO:0005125 | | cytokine activity | 235/18410 | 0.000512 | 0.000224 | 5 |
| MF | GO:0019199 | | transmembrane receptor protein kinase activity | 79/18410 | 0.000295 | 0.000129 | 4 |
| MF | GO:0019838 | | growth factor binding | 139/18410 | 0.000569 | 0.000249 | 4 |
| MF | GO:0005126 | | cytokine receptor binding | 272/18410 | 0.004153 | 0.001818 | 4 |

GO,Gene Ontology; BP,biological process; CC,Cell Component; MF,Molecular Function; KEGG,Kyoto Encyclopedia of Genes and Genomes.

**Fig.S3**

**
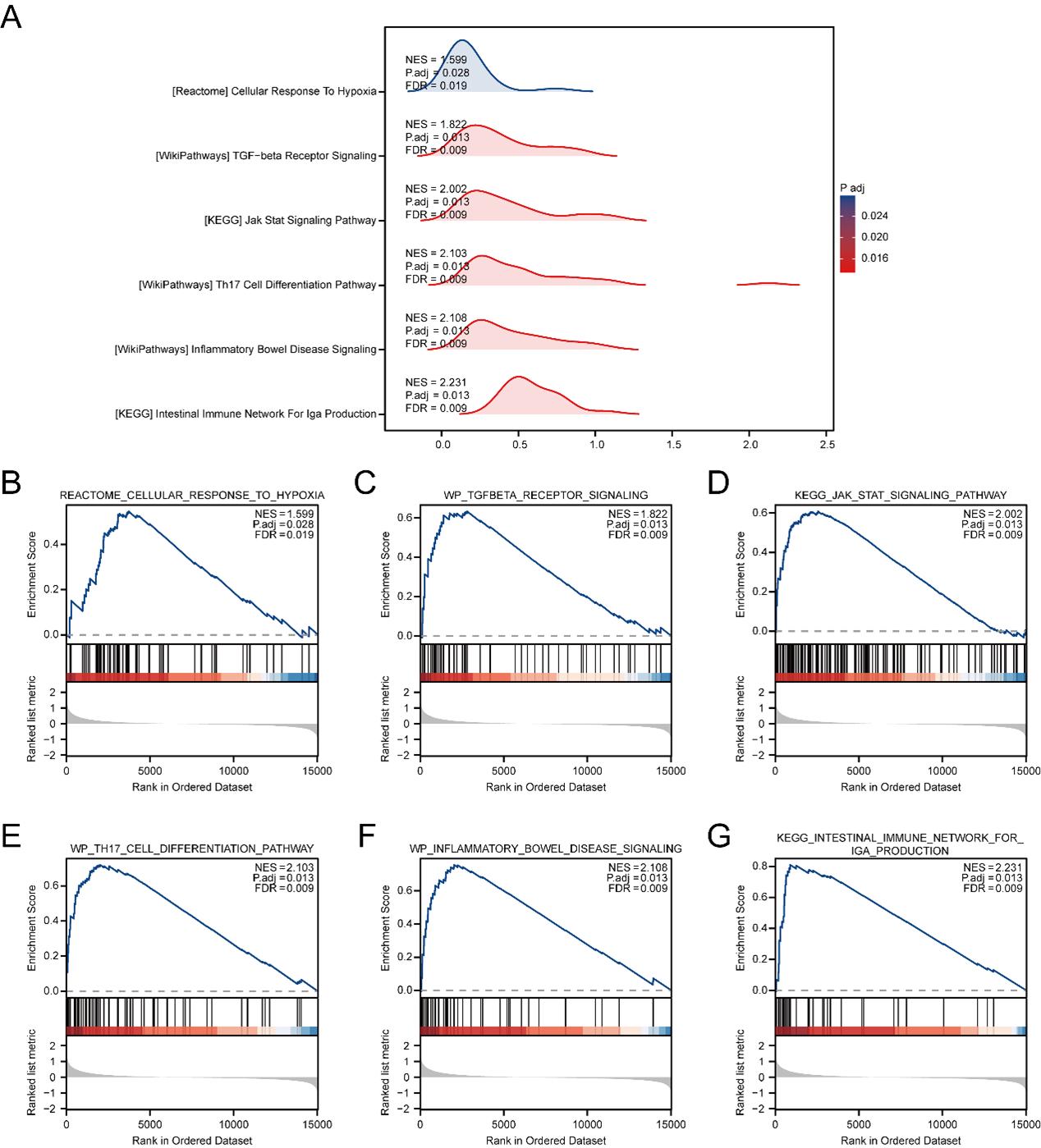
**

**Table S6. Combined Datasets analysis of GSEA between IBD and Control Groups.**

| ID | setSize | enrichmentScore | NES | p.adjust | qvalue | rank |
| --- | --- | --- | --- | --- | --- | --- |
| WP_INFLAMMATORY_BOWEL_DISEASE_SIGNALING | 43 | 0.744843 | 2.120315 | 0.015012 | 0.01045 | 2249 |
| WP_CYTOKINES_AND_INFLAMMATORY_RESPONSE | 24 | 0.840635 | 2.048222 | 0.015012 | 0.01045 | 1038 |
| KEGG_JAK_STAT_SIGNALING_PATHWAY | 140 | 0.583926 | 1.966482 | 0.015012 | 0.01045 | 2502 |
| WP_IL6_SIGNALING_PATHWAY | 43 | 0.664187 | 1.890715 | 0.015012 | 0.01045 | 1243 |
| WP_MAPK_SIGNALING_PATHWAY | 232 | 0.399101 | 1.411121 | 0.035522 | 0.024727 | 2686 |
| WP_TGFBETA_RECEPTOR_SIGNALING | 51 | 0.67489 | 1.97685 | 0.015012 | 0.01045 | 2118 |

GSEA: Gene Set Enrichment Analysis; IBD,Inflammatory Bowel Disease.

**Table S7. Combined Datasets analysis of GSEA between High Risk and Low Risk Groups.**

| ID | setSize | enrichmentScore | NES | p.adjust | qvalue | rank |
| --- | --- | --- | --- | --- | --- | --- |
| KEGG_INTESTINAL_IMMUNE_NETWORK_FOR_IGA_PRODUCTION | 40 | 0.808679 | 2.231091 | 0.013372 | 0.009216 | 899 |
| WP_INFLAMMATORY_BOWEL_DISEASE_SIGNALING | 43 | 0.755322 | 2.107726 | 0.013372 | 0.009216 | 2035 |
| WP_TH17_CELL_DIFFERENTIATION_PATHWAY | 66 | 0.719195 | 2.103305 | 0.013372 | 0.009216 | 2020 |
| KEGG_JAK_STAT_SIGNALING_PATHWAY | 140 | 0.608278 | 2.001765 | 0.013372 | 0.009216 | 2569 |
| WP_TGFBETA_RECEPTOR_SIGNALING | 51 | 0.633454 | 1.822492 | 0.013372 | 0.009216 | 2812 |
| REACTOME_CELLULAR_RESPONSE_TO_HYPOXIA | 65 | 0.546364 | 1.599106 | 0.027867 | 0.019205 | 3732 |

GSEA: Gene Set Enrichment Analysis.

**Fig.S1**

**
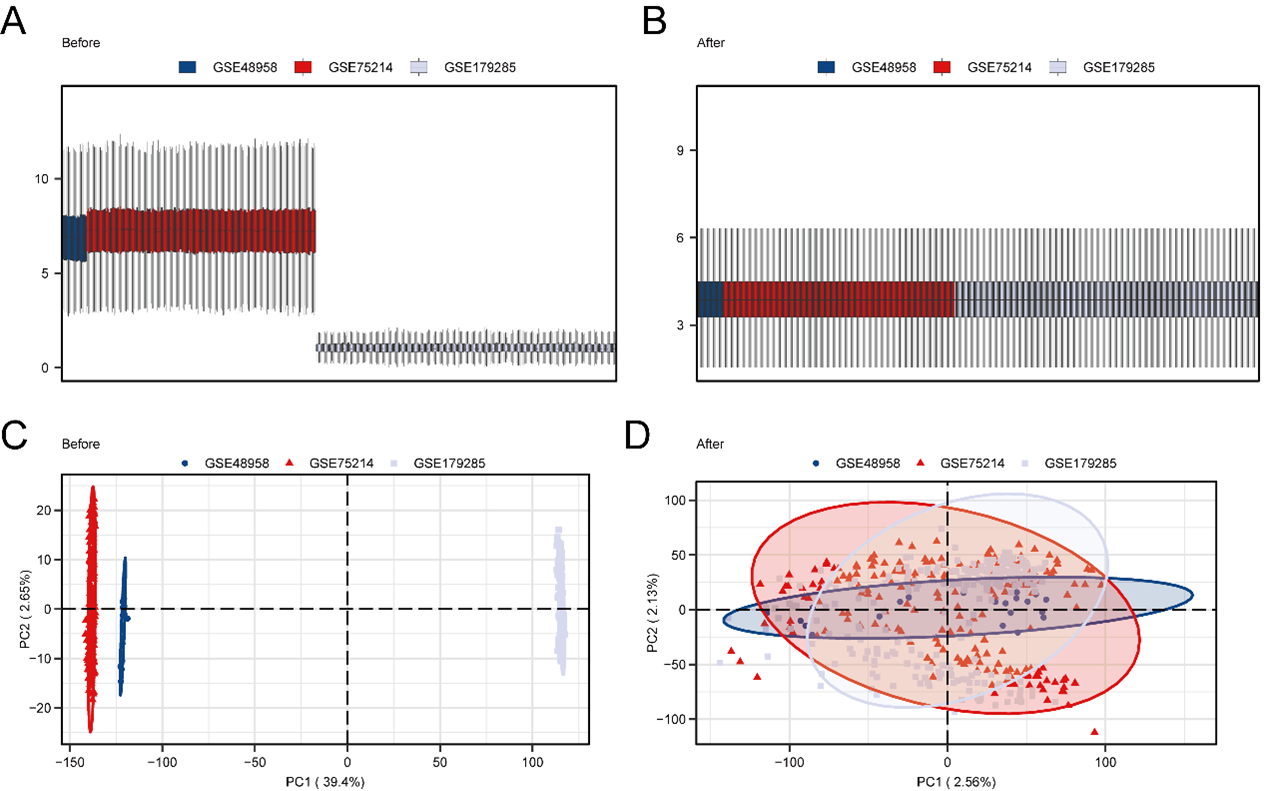
**

**Fig.S2**

**
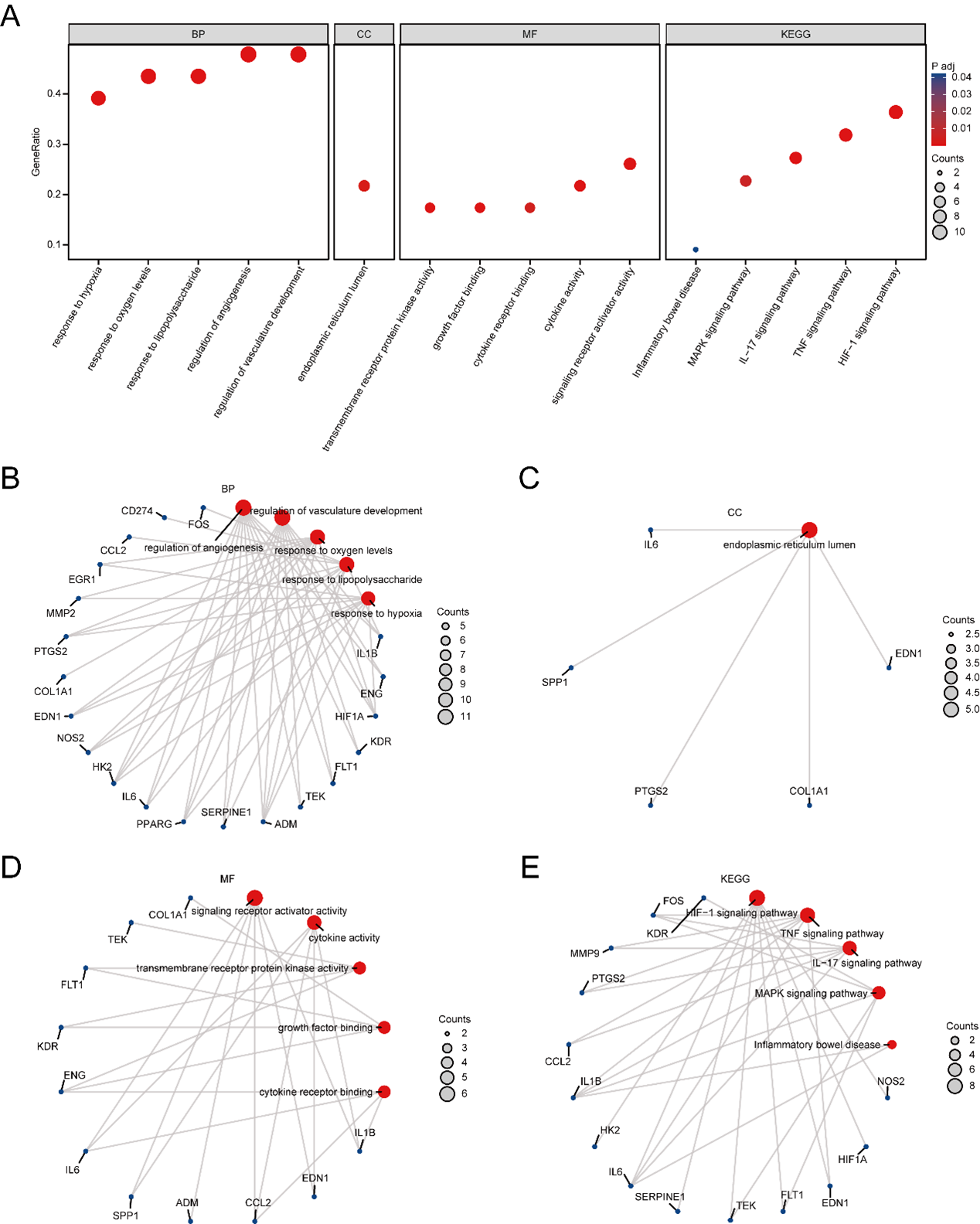
**
